# Meta-analysis under imbalance in measurement of confounders in cohort studies using only summary-level data

**DOI:** 10.1186/s12874-022-01614-9

**Published:** 2022-05-19

**Authors:** Debashree Ray, Alvaro Muñoz, Mingyu Zhang, Xiuhong Li, Nilanjan Chatterjee, Lisa P. Jacobson, Bryan Lau

**Affiliations:** 1 Department of Epidemiology, Bloomberg School of Public Health, Johns Hopkins University, Baltimore, Maryland USA; 2 Department of Biostatistics, Bloomberg School of Public Health, Johns Hopkins University, Baltimore, Maryland USA; 3 Department of Oncology, School of Medicine, Johns Hopkins University, Baltimore, Maryland USA

**Keywords:** Bias, Collective analysis, Confounder imbalance, Data integration, Meta-analysis, Omitted covariate, Omitted variable bias, Regression estimates

## Abstract

**Background:**

Cohort collaborations often require meta-analysis of exposure-outcome association estimates across cohorts as an alternative to pooling individual-level data that requires a laborious process of data harmonization on individual-level data. However, it is likely that important confounders are not all measured uniformly across the cohorts due to differences in study protocols. This imbalance in measurement of confounders leads to association estimates that are not comparable across cohorts and impedes the meta-analysis of results.

**Methods:**

In this article, we empirically show some asymptotic relations between fully adjusted and unadjusted exposure-outcome effect estimates, and provide theoretical justification for the same. We leverage these results to obtain fully adjusted estimates for the cohorts with no information on confounders by borrowing information from cohorts with complete measurement on confounders. We implement this novel method in CIMBAL (confounder imbalance), which additionally provides a meta-analyzed estimate that appropriately accounts for the dependence between estimates arising due to borrowing of information across cohorts. We perform extensive simulation experiments to study CIMBAL’s statistical properties. We illustrate CIMBAL using National Children’s Study (NCS) data to estimate association of maternal education and low birth weight in infants, adjusting for maternal age at delivery, race/ethnicity, marital status, and income.

**Results:**

Our simulation studies indicate that estimates of exposure-outcome association from CIMBAL are closer to the truth than those from commonly-used approaches for meta-analyzing cohorts with disparate confounder measurements. CIMBAL is not too sensitive to heterogeneity in underlying joint distributions of exposure, outcome and confounders but is very sensitive to heterogeneity of confounding bias across cohorts. Application of CIMBAL to NCS data for a proof-of-concept analysis further illustrates the utility and advantages of CIMBAL.

**Conclusions:**

CIMBAL provides a practical approach for meta-analyzing cohorts with imbalance in measurement of confounders under a weak assumption that the cohorts are independently sampled from populations with the same confounding bias.

**Supplementary Information:**

The online version contains supplementary material available at (10.1186/s12874-022-01614-9).

## Background

In cohort collaborations, such as the Environmental influences on Child Health Outcomes (ECHO), analyses are often done by pooling cohort-level results using meta-analysis techniques [1]. We use the term ‘collective analysis’ to describe collaborative meta-analysis in which an *apriori* analytical plan is developed to answer a specific research question, the analytic code is developed centrally, distributed to the individual cohorts, applied to each cohort’s data, and results are collected from each cohort which are then meta-analyzed. This increases uniformity with the idea of reducing bias. Further, due to onerous computational needs of big data– as is common in modern observational epidemiologic studies and genome-wide association studies–, divide-and-conquer approaches are becoming popular. A divide-and-conquer algorithm involves dividing the data into independent blocks, analyzing each block separately, and combining solutions from each block to get the solution for the full data [2]. If the goal is to estimate model parameters from the full data, then meta-analysis can be considered a divide-and-conquer approach that avoids compute-intensive model fitting on a very large sample size. Several cohort collaborations such as the Chronic Kidney Disease Prognosis Consortium (CKD-PC) operate exclusively in this manner [3].

Despite practical advantages of meta-analysis in cohort collaborations, it is likely that important confounders are not necessarily measured across all cohorts since each cohort may have been independently funded with independent study protocols. We refer to this problem as ‘confounder imbalance’. As pointed by Voils et al. [4], there appears to be no consensus on how to synthesize adjusted and unadjusted parameter estimates in a meta-analysis. Therefore, it is an open question of how to deal with this situation in the collective analysis. The simplest option is to combine unadjusted estimates only because it is available from all studies and is easy to interpret. Historically, unadjusted analyses have been emphasized for their interpretability and generalizability [5]. However, unadjusted estimates provide biased inference about the exposure-outcome association. The investigator could also be most conservative by restricting the analysis to cohort studies which have measured all pertinent confounders. This is likely to result in decreased sample size (and hence loss of information) that these cohort collaborations often capitalize upon. Alternatively, as the CKD-PC has done previously [6], the cohort collaboration may list deviations such as not having measured particular variables in an appendix. This potentially combines various estimands in which some are adjusted for all possible confounders and others for only a subset of confounders, thus ignoring potential heterogeneity in estimates. Yet another approach is to conduct separate meta-analysis of unadjusted and adjusted estimates, report both estimates, and qualitatively assess the conclusions from each. This may lead to difficulty in interpretation if the two meta-analysis results do not support the same conclusion. These naive approaches have been described with examples elsewhere [4].

A more sophisticated and statistically principled approach is GENMETA, a generalized meta-analysis method that relies on an external reference dataset to provide information on the joint distribution of covariates that are needed for the analysis [7]. The reference data can be of fairly modest size, should be independent of the cohorts under study, should have individual-level measurements on all possible covariates, and need not be linked to the outcome of interest. Using GENMETA, one can capitalize on this external data source to correct for missing confounders by relying on the joint distribution as a mapping to allow for fully adjusted estimates. However, an external data source representative of the underlying population from which the cohort studies are being drawn may not always be available. Kundu and Chatterjee [8] subsequently relaxed this independence assumption for the external data source in GENMETA.

In this article, using the idea of ‘confounding risk ratio’ (ratio of the unadjusted risk ratio to the adjusted risk ratio) [9, 10], we show how information may be borrowed across cohorts or studies reporting only summary-level data to result in fully adjusted estimates to be combined in the meta-analysis step. Our approach enables cohorts with incomplete information on confounders to contribute to the collective analysis. Our focus is on meta-analysis in the setting of parametric regression modelling of exposure-outcome association given a set of confounders. Although we describe our approach in the context of cohort studies, it is also relevant for a meta-analysis of randomized controlled trials, where the imbalance in measuring the effect modifiers across trials is prevalent. We first empirically observe that for a large enough cohort the difference of the exposure-outcome effect estimates (fully adjusted vs unadjusted) is independent of the sample size for both linear and logistic regressions. Using a generalized linear model, we theoretically justify that this limiting behavior is not unexpected. We additionally provide exact theoretical limits in the linear regression framework. We then leverage these asymptotic relations to implement our approach in a novel meta-analysis method for confounder imbalance, which we refer to as CIMBAL (implementation available in R, https://github.com/RayDebashree/cimbal). We provide details on how CIMBAL not only imputes the adjusted estimates (effect estimates and their variances) for cohorts with missing confounders but also provides a meta-analyzed estimate that appropriately accounts for the dependence between estimates arising due to borrowing of information across cohorts. We perform extensive simulation experiments to empirically demonstrate the afore-mentioned asymptotic relations under different generalizations, and to study statistical properties such as bias, type I error and power of CIMBAL. To illustrate the application of CIMBAL, we present a proof-of-concept meta-analysis of randomly chosen subsets of the National Children’s Study (NCS) data to estimate association of maternal education and low birth weight in infants, adjusting for maternal age at delivery, race/ethnicity, marital status, and income.

## Methods

### Notation, models, and existing approaches

Consider the following measurements from an epidemiologic study: response or outcome *Y*, exposure *X* and the full set of *q* possible confounders $\boldsymbol {\mathcal {C}}$. Our interest lies in quantifying the true exposure-outcome association. To assess exposure-outcome association, a cohort with no information on any confounder will consider an unadjusted model

$$\begin{aligned} Y =& \alpha_{\text{unadj}} + \beta_{\text{unadj}} X+ \varepsilon_{u}, \:\\ &\varepsilon_{u}\sim N\left(0,\sigma_{\text{unadj}}^{2}\right)\!\! \quad({\text{if continuous response}}) \\ \text{logit}\left(P(Y=1)\right) =& \alpha_{\text{unadj}} + \beta_{\text{unadj}} X \qquad({\text{if binary outcome})} \end{aligned} $$

and report unadjusted estimate of association ($\hat {\beta }_{\text {unadj}}$) and its standard error (SE) ($\hat {\text {se}}_{\text {unadj}}$). On the other hand, a cohort that measured all relevant confounders will consider a fully adjusted model

$$\begin{aligned} Y =& \alpha_{\text{adj}} + \beta_{\text{adj}} X + \boldsymbol{\gamma}'\boldsymbol{\mathcal{C}} + \varepsilon_{a}, \: \\ &\varepsilon_{a}\sim N\left(0,\sigma_{\text{adj}}^{2}\right)\ \quad({\text{if continuous response}}) \\ \text{logit}\left(P(Y=1)\right) =& \alpha_{\text{adj}} + \beta_{\text{adj}} X + \boldsymbol{\gamma}'\boldsymbol{\mathcal{C}}\ \quad({\text{if binary outcome}}) \end{aligned} $$

and report adjusted estimate of association ($\hat {\beta }_{\text {adj}}$) and its SE ($\hat {\text {se}}_{\text {adj}}$). For simplicity, in this article we will focus on cohorts with either no or full confounder information. Let us assume there are *K* cohorts, of which *K*_*c*_ cohorts have complete information on confounders while *K*_*m*_ cohorts have no confounder information. Thus, one can gather the unadjusted estimates of exposure-outcome association from all cohorts $\left \{ \left (\hat {\beta }_{j,\text {unadj}}, \hat {\text {se}}_{j,\text {unadj}}\right),\; j=1,2,...,K \right \}$ and the adjusted estimates from the complete cohorts $\left \{ \left (\hat {\beta }_{j,\text {adj}}, \hat {\text {se}}_{j,\text {adj}}\right),\; j=1,2,...,K_{c} \right \}$.

**Meta-analysis: unadjusted.** This is the most common meta-analysis approach for combining parameter estimates from studies with disparate sets of confounders. The fixed-effect inverse-variance weighted meta-analysis estimate and its SE are given by $\hat {\beta }_{\text {unadj}}^{(\text {meta})} = \frac {\sum _{j=1}^{K} w_{j} \hat {\beta }_{j,\text {unadj}}}{\sum _{j=1}^{K} w_{j}} $ and $\hat {\text {se}}_{\text {unadj}}^{(\text {meta})} = \frac {1}{\sqrt {\sum _{j=1}^{K} w_{j}}}$, where $w_{j} = 1/\hat {\text {se}}_{j,\text {unadj}}^{2}$ for all *j*=1,2,...,*K*.

**Meta-analysis: complete only.** Since unadjusted estimates are biased and usually artificially large in the presence of unmeasured confounders, this approach only combines the studies with fully adjusted estimates. The meta-analyzed effect estimate and its SE are given by $\hat {\beta }_{\text {adj}}^{(\text {meta})} = \frac {\sum _{j=1}^{K_{c}} w_{j} \hat {\beta }_{j,\text {adj}}}{\sum _{j=1}^{K_{c}} w_{j}} $ and $\hat {\text {se}}_{\text {adj}}^{(\text {meta})} = \frac {1}{\sqrt {\sum _{j=1}^{K_{c}} w_{j}}}$, where $w_{j} = 1/\hat {\text {se}}_{j,\text {adj}}^{2}$ for all *j*=1,2,...,*K*_*c*_.

### Idea of confounding risk ratio

Assuming a binary outcome *Y*, a binary exposure *X* and a binary unmeasured confounder *C*, Cornfield et al. [11] showed the following inequalities must hold in order for the confounder to fully explain the observed exposure-outcome association:

$$\begin{array}{*{20}l} \text{RR}_{XY}\leqslant \text{RR}_{CY} \text{ and } \text{RR}_{XY}\leqslant p_{1}/p_{0} \end{array} $$

where RR_*XY*_ is the risk ratio for the association between the outcome and the exposure, RR_*CY*_ is the risk ratio for the association between the outcome and the confounder, and *p*_0_ (*p*_1_) is the prevalence of the confounder in the unexposed (exposed) group. Later, Flanders and Khoury [9] proposed the idea of confounding risk ratio (coRR) [12] to quantify unmeasured confounding, and determined the bounds of coRR as

$$\begin{array}{*{20}l} 1 \leqslant \text{coRR} \leqslant \min\{\text{OR}_{XC}, \text{RR}_{CY}, 1/p_{0}, \text{RR}_{CY}/(1-p_{0} \\+ p_{0} \text{RR}_{CY}), \text{OR}_{XC}/(1-p_{0} + p_{0} \text{OR}_{XC})\} \end{array} $$

where OR_*XC*_ is the odds ratio for the association of the exposure with the confounder. In an analysis of a single cohort without access to other information about the unmeasured confounder *C*, an investigator would be required to make estimates of these parameters from some source (e.g., from literature or expert opinion) to define the bounds. However, in a cohort collaboration where one or more cohorts have collected the pertinent set of confounders, this information may be used and applied to the cohorts that did not measure one or more of the confounders. In other words, the cohorts with a complete set of confounders may provide the coRR that may be applied to crude (unadjusted or partially adjusted) estimates of association. We build on this idea to provide fully adjusted estimates of exposure-outcome association for cohorts with no information on confounders using fully adjusted and unadjusted estimates from cohorts with complete information on confounders. We empirically show asymptotic relations between adjusted and unadjusted estimates from both linear (continuous response) and logistic (binary outcome) regressions, and also provide theoretical support for these relations in a generalized linear model setup.

### Relations between adjusted and unadjusted estimates and their variances

#### Linear regression

Using simulations on a continuous response, a binary or a continuous exposure and two binary confounders, we observe that the difference of association estimates $\hat {\beta }_{\text {unadj}} - \hat {\beta }_{\text {adj}}$ (commonly referred to as ‘omitted variable bias’ in econometrics literature) is stabilizing to a constant as sample size increases (Supplementary S1). This appears to be true regardless of the strength and direction of the true exposure-outcome association.

To theoretically explore this relation, we assume all the variables *Y*, *X* and $\boldsymbol {\mathcal {C}}$ are continuous, and that the following joint distribution of variables holds at the population-level:

$$\begin{aligned} Y =& \alpha_{\text{adj}} + \beta_{\text{adj}} X + \boldsymbol\gamma'\boldsymbol{\mathcal{C}} + \varepsilon_{a}, \text{ where} ~~ \\ &\varepsilon_{a}\sim\! N\left(0,\sigma_{\text{adj}}^{2}\right)\! \qquad ({\text{True model}})\\ X =& \eta_{0} + \boldsymbol{\eta}'\boldsymbol{\mathcal{C}} + \varepsilon_{x}, \text{ where}~~ \varepsilon_{x} \sim N\left(0,\sigma_{x}^{2}\right) \\ \boldsymbol{C} =& \boldsymbol{\mu} + \boldsymbol{\varepsilon}_{c}, \text{where} ~~\boldsymbol{\varepsilon}_{c} \sim N(\boldsymbol{0},\boldsymbol{\Omega}) \end{aligned} $$

A cohort, which randomly sampled *n* individuals from this population and measured *Y*, *X* and $\boldsymbol {\mathcal {C}}$ will consider a fully adjusted model to determine exposure-outcome association using ($\hat {\beta }_{\text {adj}}, \hat {\text {se}}_{\text {adj}}^{2}$). It may also consider an unadjusted model without any confounder adjustment and get ($\hat {\beta }_{\text {unadj}}, \hat {\text {se}}_{\text {unadj}}^{2}$). Note that in the population (true model) all the variables *Y*, *X* and $\boldsymbol {\mathcal {C}}$ are considered random. In the sample (adjusted or unadjusted model), *Y* is treated as random while *X* and $\boldsymbol {\mathcal {C}}$ are assumed to be fixed. For simplicity of theoretical exposition, we assume *α*_unadj_=0=*α*_adj_, which is satisfied when the variables in the models are centered around their means. Then, the relation between the unadjusted and the adjusted effect estimates from linear regression is given by the following result.

##### **Result 1**

Under the probability law (true model) assumed above,

$$\begin{array}{*{20}l} &\hat{\beta}_{\text{unadj}} - \hat{\beta}_{\text{adj}} \quad {\overset{P}\longrightarrow} \quad \frac{\boldsymbol{\eta}'\boldsymbol{\Omega} \boldsymbol\gamma}{\sigma_{x}^{2} + \boldsymbol{\eta}'\boldsymbol{\Omega}\boldsymbol{\eta}} \text{ as } n \rightarrow \infty \end{array} $$

where $\hat {\beta }_{\text {unadj}}$ and $\hat {\beta }_{\text {adj}}$ are obtained from linear regression models of *Y* on *X* unadjusted and adjusted for confounders respectively, and $\overset {P}{\longrightarrow }$ denotes convergence in probability.

In other words, for a large enough cohort, the difference of the unadjusted and the adjusted effect estimates from a linear regression model are independent of the true exposure-outcome effect size (*β*_adj_) and also of the sample size (*n*).

##### **Result 2**

Under the true model assumed above, the effect estimates from a linear regression model are non-negatively correlated, i.e., $\text {Cov}(\hat {\beta }_{\text {unadj}}, \hat {\beta }_{\text {adj}})\geq 0$.

Proofs of these results are outlined in Supplementary S2. We also provide empirical proof of Result 1 by simulating data from the same data generating model as the population-level true model assumed above (Supplementary S2). For more general settings, such as non-normal distributions for the exposure and the confounders, our empirical evidence supports that $\hat {\beta }_{\text {unadj}} - \hat {\beta }_{\text {adj}}$ is asymptotically independent of true exposure-outcome association and the sample size (Supplementary S1). It is quite possible that these results are not new since many researchers across different quantitative fields have done theoretical work on linear models for several years [13, 14]. Notwithstanding this possibility, we state them here for completeness as these relations are leveraged by our novel meta-analysis approach.

#### Logistic regression

Using simulations, the above asymptotic sample size invariance property appears to hold for a logistic regression with binary outcome *Y*, any exposure *X* and any confounders $\boldsymbol {\mathcal {C}}$ (Supplementary S3). In other words, for a large enough cohort, $\hat {\beta }_{\text {unadj}} - \hat {\beta }_{\text {adj}} \stackrel {\text {\tiny def}}{=} \log (\hat {\text {OR}}_{\text {unadj}}) - \log (\hat {\text {OR}}_{\text {adj}})$ does not appear to depend on the sample size. Unlike the linear regression scenario, this relation does not appear to be independent of the true odds ratio ($\phantom {\dot {i}\!}\text {OR}_{\text {adj}} = e^{\beta _{\text {adj}}}$) in logistic regression. In fact, under certain assumptions– including binary exposure *X*, independence of *X* and $\boldsymbol {\mathcal {C}}$ as in a randomized trial, and weak effects ***γ*** of covariates $\boldsymbol {\mathcal {C}}$–, Gail et al. [15] derived the asymptotic approximate bias $\hat {\beta }_{\text {unadj}} - \hat {\beta }_{\text {adj}}$ for non-linear models using second order Taylor series. In particular for the logistic model, they found this bias to depend on the true exposure-outcome association in their numerical studies: $\hat {\beta }_{\text {unadj}}$ is negatively biased when *β*_adj_>0 and positively biased for *β*_adj_<0. Note, this conclusion about direction of bias was strictly based on small values of $\frac {1}{4}\boldsymbol \gamma '\boldsymbol {\Omega }\boldsymbol \gamma $, where ***γ*** consists of covariate effects in the fully adjusted model and ***Ω*** is the covariate variance-covariance matrix [15].

#### Generalized linear model

The following result generalizes the above asymptotic sample size invariance property for generalized linear models using asymptotic normality of maximum likelihood estimates.

##### **Result 3**

For a large enough cohort, the difference of the unadjusted and the adjusted effect estimates, $\hat {\beta }_{\text {unadj}} - \hat {\beta }_{\text {adj}}$, from a generalized linear model is asymptotically constant (independent of the sample size).

The proof is outlined in Supplementary S4.

### CIMBAL: proposed approach to adjust for confounder imbalance

#### Assumptions

For the ease of exposition, we will first consider only two cohorts and later generalize our approach for multiple cohorts. CIMBAL relies on asymptotic Result 3 and depends on a weak assumption that the cohorts are drawn independently from populations with the same confounding bias regardless of other types of heterogeneity (e.g., different distributions of confounders across cohorts). As will become evident in the following sections, CIMBAL does not depend on strong assumptions such as cohorts drawn from the same underlying population or homogeneity of joint distributions $[Y,X,\boldsymbol {\mathcal {C}}]$ underlying each cohort.

#### Imputed adjusted effect estimate

If there are two independent cohorts– cohort 1 with no information on any confounder and is able to report only $\hat {\beta }_{\text {unadj}}$, and cohort 2 with complete information to be able to report both $\hat {\beta }_{\text {adj}}$ and $\hat {\beta }_{\text {unadj}}$– the investigator can impute the adjusted association estimate for the cohort with missing confounder information leveraging Result 3:

1 $$ \tilde\beta_{1,\text{adj}} = \hat{\beta}_{2,\text{adj}} - \hat{\beta}_{2,\text{unadj}} {+} \hat{\beta}_{1,\text{unadj}}   $$

However, for meta-analysis, having the adjusted effect estimates is not enough. The SE of the effect estimate from the fully adjusted model is required from all the cohorts for inverse variance weighting and for obtaining the SE and the 95% confidence interval (CI) of the meta-analyzed effect.

#### Imputed adjusted standard error estimate

From Eq. 1, the adjusted variance estimate for cohort 1 may be obtained as

2 $$ \begin{aligned} \tilde{se}^{2}_{1,\text{adj}} = \hat{\text{se}}^{2}_{2,\text{adj}} + \hat{\text{se}}^{2}_{2,\text{unadj}} + \hat{\text{se}}^{2}_{1,\text{unadj}} - 2\text{Cov}(\hat{\beta}_{2,\text{unadj}},\hat{\beta}_{2,\text{adj}}) \\ \end{aligned}  $$

We call our proposed correction approach for confounder imbalance CIMBAL.

#### Meta-analysis using imputed estimates under confounder imbalance

To explain meta-analysis in this context, we continue discussion with cohort 1, which has the imputed adjusted estimates ($\tilde \beta _{1,\text {adj}}, \tilde {se}^{2}_{1,\text {adj}}$) and cohort 2, which reported the fully adjusted estimates ($\hat {\beta }_{2,\text {adj}}, \hat {\text {se}}^{2}_{2,\text {adj}}$). Inverse-variance weighted fixed-effect meta-analysis is a popular approach for pooling estimates from independent cohorts. Although we assume cohort 1 to be independent of cohort 2 (i.e., no sharing of samples between cohorts), Eq. 1 indicates that the estimates $\tilde \beta _{1,\text {adj}}$ and $\hat {\beta }_{2,\text {adj}}$ are no longer uncorrelated:

$$\begin{aligned} \text{Cov}_{b}(\tilde\beta_{1,\text{adj}}, \hat{\beta}_{2,\text{adj}}) &= \text{Cov}_{b}(\hat{\beta}_{2,\text{adj}} - \hat{\beta}_{2,\text{unadj}} + \hat{\beta}_{1,\text{unadj}}, \hat{\beta}_{2,\text{adj}}) \\&= \text{Var}(\hat{\beta}_{2,\text{adj}}) - \text{Cov}(\hat{\beta}_{2,\text{unadj}},\hat{\beta}_{2,\text{adj}}) \end{aligned} $$

where Cov_*b*_(.) denotes between-cohort covariance, to differentiate from Cov(.) that captures within-cohort co-variability. Consequently, the inverse-variance weights are not optimal in terms of statistical efficiency of the meta-analyzed estimate [16, 17]. The following result gives the linear combination of exposure-outcome association estimates with the smallest asymptotic variance among all linear estimators.

##### **Result 4**

The exposure-outcome association estimate from meta-analyzing CIMBAL-imputed adjusted estimate from one cohort and the fully adjusted estimate from another cohort is given by

$$\begin{array}{*{20}l} \hat{\beta}_{\text{adj}} &= \hat{w}_{1}\tilde\beta_{1,\text{adj}} + \hat{w}_{2}\hat{\beta}_{2,\text{adj}} \end{array} $$

where the optimal weights maximizing statistical efficiency are

$$\begin{array}{*{20}l} \hat{w}_{1} &= \frac{\text{Cov}\left(\hat{\beta}_{2,\text{unadj}},\hat{\beta}_{2,\text{adj}}\right)}{{\hat{\text{se}}^{2}_{1,\text{unadj}} + \hat{\text{se}}^{2}_{2,\text{unadj}}} }\; \text{ and} \;\hat{w}_{2} = 1-\hat{w}_{1} \end{array} $$

and the corresponding meta-analyzed adjusted SE estimate is

$$\begin{array}{*{20}l} \hat{\text{se}}_{\text{adj}}&= {\sqrt{\hat{\text{se}}^{2}_{2,\text{adj}} - \frac{\text{Cov}\left(\hat{\beta}_{2,\text{unadj}},\hat{\beta}_{2,\text{adj}}\right)^{2}}{\hat{\text{se}}^{2}_{1,\text{unadj}} + \hat{\text{se}}^{2}_{2,\text{unadj}}}}} \end{array} $$

The proof is outlined in Supplementary S4.

The meta-analyzed SE estimate as well as the weights depend on $\text {Cov}\left (\hat {\beta }_{2,\text {unadj}},\hat {\beta }_{2,\text {adj}}\right)$ which does not have a closed form for logistic regression. We have theoretically shown that $\text {Cov}\left (\hat {\beta }_{2,\text {unadj}},\hat {\beta }_{2,\text {adj}}\right) \geqslant \ 0$ for linear regression (Result 2), and empirically shown that it holds for logistic regression for a large enough number of cohorts (Supplementary S3). If there are multiple cohorts with full confounder information, we can use the estimated covariance between their adjusted and unadjusted estimates as an estimate for $\text {Cov}\left (\hat {\beta }_{2,\text {unadj}},\hat {\beta }_{2,\text {adj}}\right)$. If there are insufficient number of cohorts to estimate this covariance, we suggest ignoring the covariance (i.e., assume $\text {Cov}\left (\hat {\beta }_{2,\text {unadj}},\hat {\beta }_{2,\text {adj}}\right)=0$). This will only lead to overestimation of the meta-analyzed SE or a larger CI and hence a less efficient estimate of the exposure-outcome association. The following corollary states this special case of CIMBAL.

##### **Corollary 1**

Ignoring $\text {Cov}(\hat {\beta }_{2,\text {unadj}},\hat {\beta }_{2,\text {adj}})$ in Result 4 is equivalent to taking $\hat {w}_{1}=0$, thus ignoring the study reporting only unadjusted estimates. Consequently, meta-analysis using only the adjusted estimates available from complete cohorts is a special case of CIMBAL.

#### Generalization of CIMBAL to multiple cohorts with and without confounder measurements

The proposed correction approach can be easily extended to >2 independent cohorts. If there are *K*_*c*_ cohorts with complete confounder information, we first meta-analyze these cohorts (using fixed-effect inverse-variance weighted meta-analysis) to obtain the meta-analyzed adjusted estimates and the meta-analyzed unadjusted estimates and their corresponding SE estimates: $\left (\hat {\beta }_{2,\text {adj}}^{(\text {meta})}, \hat {\text {se}}_{2,\text {adj}}^{(\text {meta})}\right)$ and $\left (\hat {\beta }_{2,\text {unadj}}^{(\text {meta})}, \hat {\text {se}}_{2,\text {unadj}}^{(\text {meta})}\right)$. Similarly, if there are *K*_*m*_ cohorts with no confounder information, we meta-analyze these cohorts to obtain the meta-analyzed unadjusted estimate and the corresponding SE estimate: $\left (\hat {\beta }_{1,\text {unadj}}^{(\text {meta})}, \hat {\text {se}}_{1,\text {unadj}}^{(\text {meta})}\right)$. Now we can apply the imputation approach using Eqs. 1 and 2 to obtain CIMBAL-imputed adjusted estimates for the pooled no-confounder cohort $\left (\tilde \beta _{1,\text {adj}}^{(\text {meta})}, \tilde {\text {se}}_{1,\text {adj}}^{(\text {meta})}\right)$. For the final meta-analysis, we first estimate $\text {Cov}\left (\hat {\beta }_{2,\text {unadj}},\hat {\beta }_{2,\text {adj}}\right)$ from the *K*_*c*_ complete cohorts and then use formulae from Result 4. We have implemented our imputation approach and the subsequent meta-analysis in a R [18] program at https://github.com/RayDebashree/cimbal.

### Simulation design

To demonstrate pitfalls of meta-analysis in cohort collaborations in the presence of unbalanced measurement of confounders, and to study the performance of our proposed approach, CIMBAL, we conduct extensive simulation experiments using a binary outcome *Y*, a binary exposure *X*, and two binary confounding variables *C*_1_,*C*_2_. Note, preliminary simulation analysis in support of asymptotic relations claimed in “Linear regression” and “Logistic regression” sections are presented in Supplementary S1 and Supplementary S3.

For a given individual, we use the model logit(*P*(*Y*=1))=*γ*_0_+*γ*_1_*C*_1_+*γ*_2_*C*_2_+*βX* to generate the outcome, where *C*_1_ and *C*_2_ are Bernoulli variables with success probabilities 0.1 and 0.6 respectively, and the exposure *X* is generated using the logistic model logit(*P*(*X*=1))=*η*_0_+*η*_1_*C*_1_+*η*_2_*C*_2_. The choices of parameters *γ*_0_,*γ*_1_,*γ*_2_,*β*,*η*_0_,*η*_1_,*η*_2_ are provided in Table 1. Setting I of parameter choices involve equal confounder effects in the same direction for both exposure and outcome (*η*_1_=*η*_2_=*γ*_1_=*γ*_2_=2). Keeping the confounder effect on exposure same, Setting II involves one confounder having equal and same effect and the other confounder having equal but opposite effect on the outcome (*η*_1_=*η*_2_=*γ*_1_=2,*γ*_2_=−2). Setting III considers equal and opposite effect of both confounders for the outcome (*η*_1_=*η*_2_=2,*γ*_1_=*γ*_2_=−2). For simplicity, we assume 60 independent cohorts of equal sample size (*n*=150) are available. We simulate 3 scenarios: (1) fewer cohorts or (2) equal number of cohorts or (3) more cohorts with no confounder information than with complete confounder information. For each scenario, we simulate 2,500 replicates of 60 cohorts. We are interested in estimating the association between the outcome and the exposure by using cohort-level summary statistics.

**Table 1:** Parameter values assumed in simulation studies. The models used for the generation of binary exposure *X* and binary outcome *Y* are respectively logit(*P*(*X*=1))=*η*_0_+*η*_1_*C*_1_+*η*_2_*C*_2_ and logit(*P*(*Y*=1))=*γ*_0_+*γ*_1_*C*_1_+*γ*_2_*C*_2_+*βX*, where confounders *C*_1_∼*Bin*(1,0.1) and *C*_2_∼*Bin*(1,0.6)

	Exposure model				Outcome model			
Setting	*η*_0_	*η*_1_	*η*_2_		*γ*_0_	*γ*_1_	*γ*_2_	*β*
I	$\log \frac {0.5}{0.5}$	2	2		$\log \frac {0.3}{0.7}$	2	2	log(1), log(3),log(1/3)
II	$\log \frac {0.5}{0.5}$	2	2		$\log \frac {0.3}{0.7}$	2	−2	log(1)
III	$\log \frac {0.5}{0.5}$	2	2		$\log \frac {0.3}{0.7}$	−2	−2	log(1)

We perform a few additional experiments to see how sensitive CIMBAL is compared to other approaches when underlying assumptions are not satisfied. In particular, for the Sensitivity I scenario, we generate all the cohorts without confounder information from the data generating model described above, and the remaining cohorts (those with complete confounder information) from another distribution so that the underlying joint distributions [*Y*,*X*,*C*_1_,*C*_2_] are not the same between complete and incomplete cohorts. The second data generating model is assumed to have different success probabilities of confounders *C*_1_ and *C*_2_ (0.2 and 0.7 respectively). This changes the mean vector as well as the variance-covariance matrix of the confounder distribution and the joint distribution [*Y*,*X*,*C*_1_,*C*_2_]. We keep the true exposure-outcome association fixed at 0 for both populations. For the Sensitivity II scenario, we consider a variation of the Sensitivity I scenario by keeping the joint distribution same across cohorts but changing the strength of confounding on *X* and *Y*. Briefly, the cohorts with no confounder information are drawn from the data generating model with strong confounding as described before, while the remaining cohorts are drawn from a data generating model with much weaker confounding effects. For the Sensitivity III scenario, we consider a third confounder *C*_3_∼*N*(0,1) in the data generating model for *Y* and *X*, where we assume *γ*_3_=2 and *η*_3_=2 are parameters corresponding to the association of *C*_3_ with *Y* and *X* respectively. For the analysis models however, we assume *C*_3_ is an unmeasured confounder so that the models fit by each cohort is either adjusted for the first two confounders (*C*_1_ and *C*_2_) or adjusted for none.

For each simulation setting and scenario, we obtain the log-odds estimate and its SE for the combined cohort using CIMBAL and compare it against two meta-analysis approaches: meta-analysis of only the available adjusted estimates, and the oracle (gold standard) meta-analysis of adjusted estimates from all cohorts. Note, we do not include meta-analysis of the unadjusted estimates from all cohorts in this comparison since it is well-established to be a biased estimate in the presence of confounders. We visually compare all three approaches by plotting the distribution of estimated log-odds and its SE. Further, we use the following metrics for comparison: mean squared error (MSE), mean width of 95% CI, and type I error (only when data are generated under *β*= log(OR)=0). For a given method, we estimate MSE as mean of the squared difference between estimated log-odds and the true log-odds; mean width as mean of the difference between the upper and the lower 95% confidence limits; and type I error as the proportion of times the null hypothesis that *β*=0 is rejected at 5% significance level. For all these metrics, average or proportion is calculated over 2,500 independent replicates. We conduct all statistical analyses in R and create plots using R package ggplot2 [19].

### Application to NCS data on low birth weight of infants

We use NCS [20, 21] data on 5,604 children enrolled between 2009 and 2013 to evaluate all the meta-analysis approaches including CIMBAL. For this proof-of-concept analysis, we study if and how maternal education influences infant birth weight-for-gestational-age (BW-for-GA). We assume the NCS dataset is our population and we randomly sample 40 independent cohorts of equal sample size from this population.

We define a dichotomized version of BW-for-GA z-score as our outcome. In particular, we extract child sex, birth weight, and gestational age from the medical records and calculate child BW-for-GA z-score according to the 2017 US reference [22]. After excluding children with missing sex, birth weight and/or gestational age at birth, and those with gestational age at birth <22 or >42 weeks, we calculate BW-for-GA z-score for 4,658 children. ‘Small’ for gestational age is typically defined as BW-for-GA z-score <10th percentile. However, this definition would lead to an outcome with low prevalence in our study, and combined with small sample size in each of the 40 cohorts may lead to unstable estimates. Hence, in this proof-of-concept analysis we define ‘small’ for gestational age as BW-for-GA z-score <25th percentile to mimic a relatively common outcome.

For our exposure, we consider two categories of maternal education. The reference category is ‘Some college or below’ while the other category is ‘Bachelor’s degree or above’. We consider 4 key maternal covariates (confounders) that may influence the exposure-outcome association: maternal age at delivery, race/ethnicity, marital status, and annual household income. While maternal age is a continuous variable, the others are categorical. We consider 4 categories for race/ethnicity: Hispanic, non-Hispanic White, non-Hispanic Black, and non-Hispanic other; 3 categories for marital status: married or living together with a partner, never been married, and divorced, separated, or widowed; 4 categories for annual household income: <$30,000, $30,000-$49,999, $50,000-$99,999, and >$100,000. After removing observations with missing confounders, we have 4,089 mother-infant pairs in our final analytical dataset.

We draw a random sample of 4,080 dyads without replacement and split them into 40 cohorts of equal sample size (*n*=102). Of the 4,080 dyads, 22.6*%* (*n*=921) of the infants had BW-for-GA z-score <25th percentile. The prevalence of our outcome in the 40 independent cohorts ranged from 12.7*%* (*n*=13) to 31.4*%* (*n*=32). For all cohorts, we obtain unadjusted as well as adjusted estimates of exposure-outcome association along with their SE estimates using Stata [23]. We also combine all the cohorts and conduct a pooled analysis without and with confounder adjustment, giving us unadjusted as well as adjusted estimates for the combined cohort. To evaluate CIMBAL in comparison to the other meta-analysis approaches described before, we assume that either 10, 20 or 30 cohorts have no information on any confounders, while the rest have complete information on all confounders. We randomly select the cohorts assumed to have no confounder information. Before meta-analyzing all the cohorts, we exclude any outlying cohorts. Specifically, if log-odds estimate or SE estimate (unadjusted and/or adjusted) from a cohort falls outside the 3 times inter-quartile range, then we exclude that cohort. We remove one such outlier, leaving 39 independent cohorts from the NCS population.

## Results

### Simulated data analysis

#### Under confounder imbalance, distributions of estimates from CIMBAL are closer to oracle than when restricted to cohorts with complete data

**Fig. 1:**
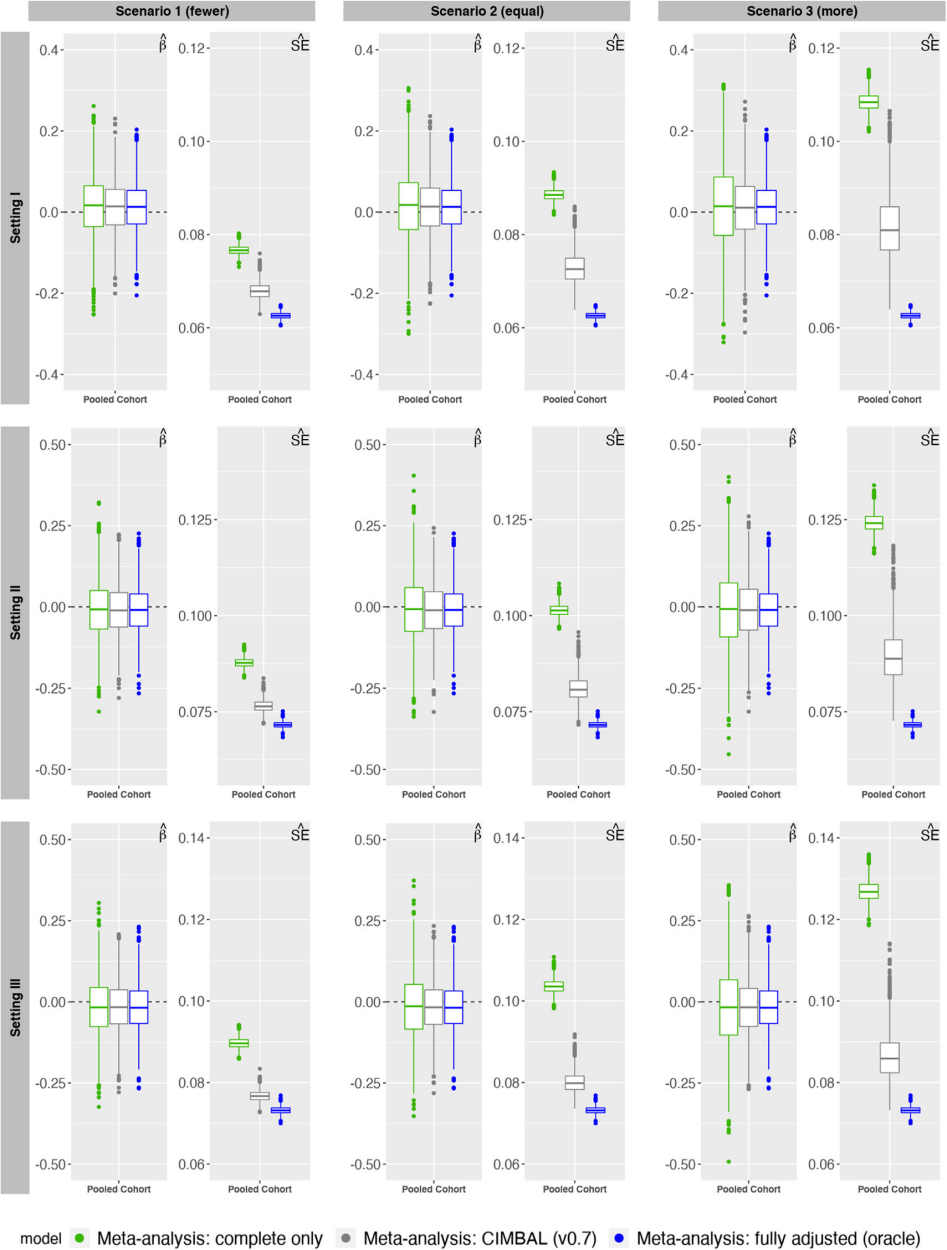
Comparison of CIMBAL with complete case meta-analysis approach and gold standard (oracle) approach across different simulated data scenarios. The log-odds estimate of the exposure-outcome association ($\hat {\beta }=\log (\hat {\text {OR}})$) and its SE $\left (\hat {\text {se}}=\sqrt {\hat {\text {Var}}(\hat {\beta })} \right)$ from the combined cohort over 2500 independent replicate datasets are plotted for each scenario: (1) fewer cohorts or (2) equal number of cohorts or (3) more cohorts with no confounder information than with complete confounder information. The horizontal dashed line in the $\hat {\beta }$-plots correspond to the true *β*=0

Figure 1 shows the distribution of the estimate of exposure-outcome association ($\hat {\beta }$) and its SE ($\hat {\text {se}}$) across different simulation scenarios. Although meta-analysis using adjusted estimates from cohorts with complete information provides unbiased estimates, it has high variability due to small effective sample size, leading to wide CIs. CIMBAL, on the other hand, provides not only unbiased estimates but also smaller variability than complete-only meta-analysis, leading to point estimates and CIs that are closest to the oracle.

Note that the current simulation model assumes strong confounder effects only, and that there is no association between the exposure and the outcome (*β*= log(1)). Therefore, we additionally simulate Setting I with weak confounder effects (Figure S7), and Setting I with strong positive (*β*= log(3)) or negative (*β*= log(1/3)) exposure-outcome association (Figure S8). We observe that relative behavior of the methods is the same regardless of the true association, the strength of the confounding effects, and whether we have fewer, equal or more cohorts with missing confounder information (Fig. 1, S7 and S8). The variability of the exposure-outcome estimate from each meta-analysis method increases as the number of cohorts with missing confounders increases. Similar behavior is observed for other simulation settings involving varying directions of confounder effects (Fig. 1). In other words, meta-analysis using CIMBAL seems to provide estimates that are closest to what one would have obtained if fully adjusted estimates were available from all cohorts.

#### Meta-analysis using CIMBAL is closest to oracle across multiple statistical metrics

Table 2 shows MSE (along with relative MSE compared to the oracle meta-analysis), mean width of 95% CI, and type I error performance of each method across different simulation scenarios. Across all parameter settings and scenarios, MSE of CIMBAL is closest to the MSE of the oracle. Complete-only meta-analysis has the largest mean width of 95% CI that increases with increasing number of cohorts without complete confounder information, as expected. Mean width of CIMBAL’s CI is only slightly larger than the oracle. These observations continue to hold for simulations involving weaker confounder effects (Table S5). As for the type I error metric that one can evaluate only under the null (i.e., when the underlying data have no exposure-outcome association), both CIMBAL and complete-only meta-analysis maintains appropriate type I error at 5% level. CIMBAL’s type I error performance resembles that of the oracle across most scenarios (Table 2), and this continues to be true regardless of strength of confounder effects (Table S5).

**Table 2:** Evaluation of CIMBAL along with complete-only meta-analysis approach and gold standard (oracle) approach using multiple metrics across different simulated data scenarios. The metrics MSE (mean squared error), rel. MSE (relative MSE compared to oracle meta-analysis approach), mean width of 95% CI, and type I error inflation factor at 5% significance level (ratio of type I error estimate to 0.05) are estimated using 2,500 independent replicate datasets for each scenario: (1) fewer cohorts or (2) equal number of cohorts or (3) more cohorts with no confounder information than with complete confounder information. Ideal rel. MSE value is 1 × and larger values indicate departure from oracle. Ideal type I error inflation value is 1; larger than 1 indicates inflation, smaller than 1 indicates conservativeness. The underlying data generative model assumes there is no exposure-outcome association (true *β*=0)

		Scenario 1 (fewer)			Scenario 2 (equal)			Scenario 3 (more)		
	Method	MSE	mean	type I	MSE	mean	type I	MSE	mean	type I
		(rel. MSE)	width	error IF	(rel. MSE)	width	error IF	(rel. MSE)	width	error IF
**Setting I**	M: complete only	0.006 (1.5 ×)	0.30	0.98	0.007 (1.8 ×)	0.35	0.86	0.011 (2.8 ×)	0.43	0.91
	M: CIMBAL (v0.7)	0.004 (1.0 ×)	0.27	1.00	0.005 (1.3 ×)	0.29	0.92	0.006 (1.5 ×)	0.32	1.00
	M: fully adjusted (oracle)	0.004 (1 ×)	0.25	1.02	0.004 (1 ×)	0.25	1.02	0.004 (1 ×)	0.25	1.02
**Setting II**	M: complete only	0.008 (1.6 ×)	0.34	0.96	0.010 (2.0 ×)	0.40	0.94	0.015 (3.0 ×)	0.49	0.91
	M: CIMBAL (v0.7)	0.006 (1.2 ×)	0.30	1.02	0.007 (1.4 ×)	0.32	1.06	0.008 (1.6 ×)	0.35	1.02
	M: fully adjusted (oracle)	0.005 (1 ×)	0.28	0.96	0.005 (1 ×)	0.28	0.96	0.005 (1 ×)	0.28	0.96
**Setting III**	M: complete only	0.008 (1.6 ×)	0.35	0.96	0.010 (2.0 ×)	0.41	0.82	0.016 (3.2 ×)	0.50	0.89
	M: CIMBAL (v0.7)	0.006 (1.2 ×)	0.30	1.02	0.006 (1.2 ×)	0.31	1.07	0.008 (1.6 ×)	0.34	0.98
	M: fully adjusted (oracle)	0.005 (1 ×)	0.29	1.00	0.005 (1 ×)	0.29	1.00	0.005 (1 ×)	0.29	1.00

Abbreviations: IF, inflation factor; M, meta-analysis of 60 cohorts

#### Meta-analysis using CIMBAL coincides with complete-only meta-analysis when there are too few cohorts with complete confounder information

Meta-analysis of CIMBAL-imputed adjusted estimates from incomplete cohorts and adjusted estimates from complete cohorts requires an estimate of $\text {Cov}\left (\hat {\beta }_{\text {unadj}}, \hat {\beta }_{\text {adj}}\right)$. This estimated covariance not only depends on the sample sizes of the complete cohorts but also the number of such cohorts used to estimate the covariance, the strengths and directions of confounder effects and exposure-outcome association (Figure S4). We suggest that at least 20 cohorts be used for appropriately estimating this covariance. If the estimate turns out to be negative, our program cimbal automatically assumes 0 covariance (the theoretical lower limit we found for linear regression case and the empirical asymptotic lower limit we found for logistic regression). Negative covariance estimate occurs when the correlation estimate is negative, which may arise due to reasons such as insufficient number of cohorts used in the estimation process, insufficient per-cohort sample size relative to confounder adjustment, or skewed sample distribution of categorical confounders between cases and controls leading to model fit issues (Figures S5 and S6). Consistent with Corollary 1, the CIMBAL meta-analyzed estimate boils down to the estimate from complete-only meta-analysis when 0 covariance is assumed (Figure S9).

#### Sensitivity analysis

When there exists heterogeneity in the underlying joint distributions– here, complete cohorts are from one population, the remaining are from another with a different joint distribution (Sensitivity I scenario)–, CIMBAL’s estimates may be slightly biased (Figure S10) but still closer to the oracle in terms of MSE than complete-only meta-analysis (Table S4). This appears to be true regardless of whether fewer or more cohorts have no information on any confounder. When the underlying joint distributions are homogenous but the confounding bias is not the same across cohorts– here, counfounding effects are weak in complete cohorts but very strong in the others (Sensitivity II scenario)–, we see massive increase in bias (Figure S11), MSE and type I error rates (Table S4) for CIMBAL. Thus, CIMBAL is very sensitive to heterogeneity of confounding bias but not as sensitive to heterogeneity in underlying joint distributions. When there is an unmeasured confounder (Sensitivity III scenario), all the meta-analysis approaches, including the oracle, are biased as expected; however, meta-analysis using CIMBAL is again closest to the oracle (Figure S12).

### NCS data analysis

**Fig. 2:**
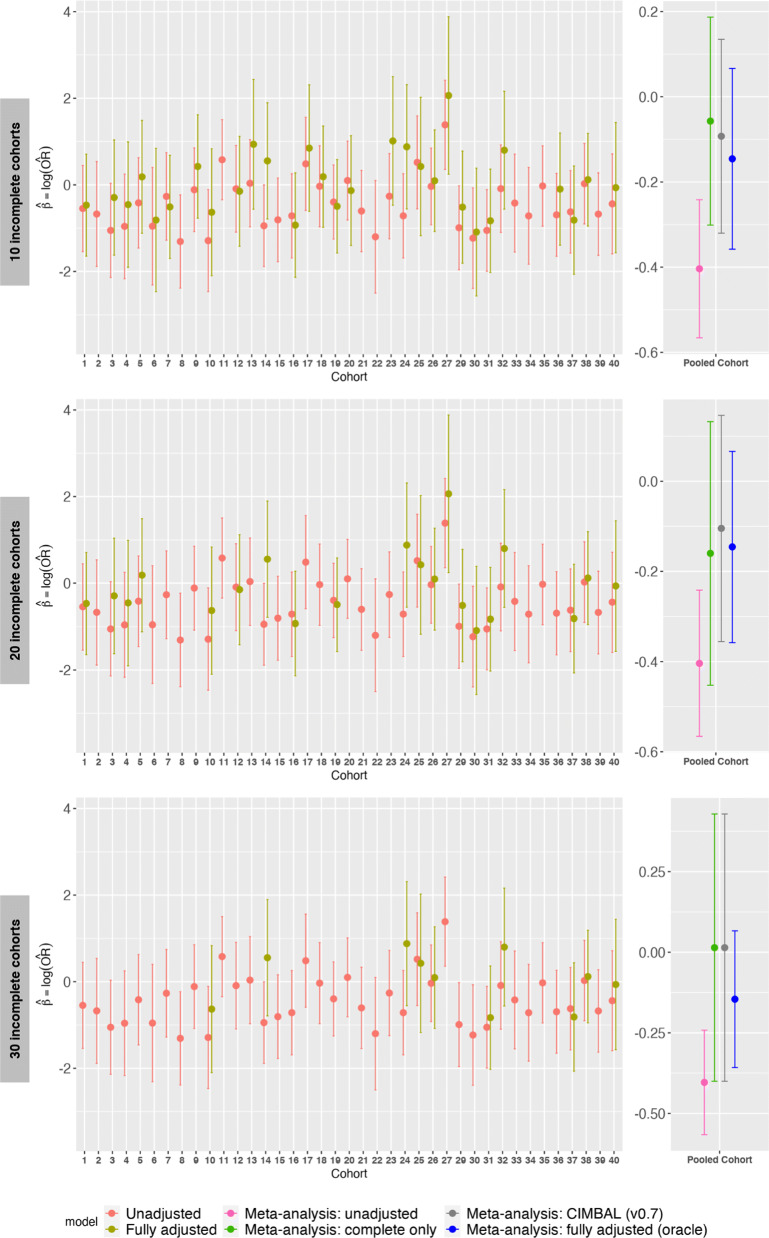
Comparison of CIMBAL with complete case meta-analysis approach and gold standard (oracle) approach in a proof-of-concept analysis using the NCS data. The log-odds estimate of the exposure-outcome association and its 95% CI are plotted

Figure 2 shows not only the reported log-odds estimate and its 95% CI from each cohort but also the different meta-analyzed estimates for the pooled cohort. For most cohorts with complete confounder information, we see appreciable difference between their adjusted and unadjusted estimates. As expected, meta-analysis using only the unadjusted estimates from all the cohorts leads to considerably biased log-odds estimate with a narrow CI around them. On the other hand, meta-analysis only using the adjusted estimates from the complete cohorts leads to less bias but larger CIs compared to the oracle. When only 10 or 20 out of 39 cohorts (1 cohort removed for being an outlier) have no confounder information, we find CIMBAL has similar bias as the complete-only meta-analysis but narrower CI. When 30 cohorts have no confounder information, we do not have enough complete cohorts to estimate the weight CIMBAL should put on incomplete cohorts and consequently we assign all the weight to complete cohorts. We see that CIMBAL and complete-only meta-analysis estimates and CIs coincide as expected.

## Discussion

In this article, we develop a novel and practical approach for meta-analysis of exposure-outcome association estimates from cohorts with disparate confounder information. Currently, there is no consensus on how unadjusted and adjusted estimates from cohorts may be meaningfully combined without sacrificing on sample size or unbiasedness of estimate. Our approach, CIMBAL, mitigates this issue by borrowing information from cohorts with complete confounder information to impute adjusted estimates for cohorts without counfounder information. This borrowing of information is grounded in asymptotic relations between adjusted and unadjusted estimates that we have justified theoretically for generalized linear models. We additionally derive the meta-analysis weights for the reported and the imputed adjusted estimates that minimizes the variance of the meta-analyzed estimate. CIMBAL is a practical approach for data integration if only summary statistics are available from cohorts. It is a generalization of meta-analysis of available adjusted estimates only, which ignores contribution from cohorts without full confounder information. When compared to popular meta-analysis approaches in the presence of confounder imbalance among cohorts, we find the CIMBAL meta-analyzed estimate to be closer to the gold standard (meta-analysis of fully adjusted estimates from all cohorts, when available) across statistical metrics such as type I error rate (for null data only), MSE, and mean-width of 95% CI. As proof of principle, we apply CIMBAL and other meta-analysis approaches to estimate association of maternal education with low birth weight of infants from NCS data after adjusting for four key maternal variables (confounders). Despite having access to individual-level data, we randomly split the NCS data into multiple cohorts, some with the necessary confounder information, others with all confounder information removed. Our real data analysis results conform with our findings from simulation experiments.

An alternative approach to circumvent missing data on confounders will be to use individual-level data from the cohorts with complete information to derive $[\boldsymbol {\mathcal {C}}|Y,X]$, the conditional distribution of the confounders given the outcome and the exposure of interest. Standard methods of multiple imputation can be applied on the cohorts with incomplete confounder information before obtaining fully adjusted estimates from them [24]. Not only does this require access to individual-level data from the cohorts with complete information but also the confounders are likely to be of large dimension making the imputation models challenging. Yet another approach is GENMETA, which too requires individual-level reference data representative of the underlying population [7, 8]. Imputing fully adjusted estimates using CIMBAL is not subjected to those requirements, and thus is a practical meta-analysis approach.

We describe CIMBAL using a fixed-effect meta-analysis framework. One could alternatively consider a random-effect meta-analysis framework, where heterogeneity among effect estimates is modeled as a variance component reflecting between-cohort variance. Specifically, one can meta-analyze unadjusted estimates from *K*_*m*_ cohorts with no confounder information using the inverse-variance weights for random-effect meta-analysis: $\omega _{j} = \frac {1}{\hat {\text {se}}_{j,\text {unadj}}^{2}+\hat \tau ^{2}}$ for *j*=1,2,...,*K*_*m*_, where $\hat \tau ^{2}$ is an estimate of between-cohort variance in $\left \{ \hat {\beta }_{j,\text {unadj}} \right \}_{j=1}^{K_{m}}$ obtained using, for instance, DerSimonian and Laird method of moments approach [25]. Alternative approaches, such as restricted maximum likelihood or non-parametric DerSimonian and Laird methods, may be used to obtain $\hat \tau ^{2}$ when method of moments gives biased estimate, as suggested by recent comparative studies [26, 27]. Similarly, the between-cohort variances in $\left \{ \hat {\beta }_{j,\text {unadj}} \right \}_{j=1}^{K_{c}}$ and $\left \{ \hat {\beta }_{j,\text {adj}} \right \}_{j=1}^{K_{c}}$ may be estimated separately, and subsequently used to obtain the inverse-variance weights for random-effect meta-analysis of the unadjusted and the adjusted estimates from *K*_*c*_ complete cohorts. This set of random-effect meta-analyzed estimates may then be used to get the CIMBAL-imputed adjusted estimates for the pooled no-confounder cohort. The final meta-analysis step (Result 4) in CIMBAL, however, needs to use a fixed-effect framework since the two estimates corresponding to two groups of cohorts are correlated and the between-group variance cannot be reliably estimated. A caveat of this random-effect framework is potentially allowing for heterogeneity of confounding bias between the complete cohorts and the incomplete ones due to differences in aspects of the underlying populations or the study designs, and CIMBAL is very sensitive to this heterogeneity.

While we demonstrate CIMBAL on cohorts with either full or no confounder information, it may also be applied to cohorts with either full or partial confounder information. Among the *q* possible confounders, suppose all cohorts have the same *p*(<*q*) confounders measured. For instance, sex and race/ethnicity information are commonly collected in epidemiologic studies. In such a scenario, one may provide the cimbal program with partially adjusted estimates instead of the unadjusted estimates. Under the hood, cimbal program first meta-analyzes all the complete cohorts to obtain $\left (\hat {\beta }_{2,\text {adj}}^{(\text {meta})}, \hat {\text {se}}_{2,\text {adj}}^{(\text {meta})}\right)$ and $\left (\hat {\beta }_{2,\text {p-adj}}^{(\text {meta})}, \hat {\text {se}}_{2,\text {p-adj}}^{(\text {meta})}\right)$, where suffix ‘p-adj’ denotes partial confounder adjustment. All the cohorts with partial confounder information are meta-analyzed to obtain $\left (\hat {\beta }_{1,\text {p-adj}}^{(\text {meta})}, \hat {\text {se}}_{1,\text {p-adj}}^{(\text {meta})}\right)$. Then, CIMBAL-imputed adjusted estimates for the pooled partial-confounder cohort are obtained as $\left (\tilde \beta _{1,\text {adj}}^{(\text {meta})}, \tilde {\text {se}}_{1,\text {adj}}^{(\text {meta})}\right)$, and meta-analyzed with the available fully adjusted estimates using Result 4.

CIMBAL is not without limitations. We make the simplifying assumption that cohorts either have full confounder information or have the same partial confounder information. By ‘full confounder information’, we mean measurements on the minimally sufficient confounder set are available to complete cohorts. However, multiple minimally sufficient adjustment sets may exist and each can be used to obtain an unbiased estimate of the exposure-outcome association. While each minimally sufficient adjustment set can, in principle, adjust for confounding, the estimands are fundamentally different. Consequently, non-collapsible association estimator like the odds ratio from a logistic regression can show substantial heterogeneity across minimally sufficient confounder sets, and meta-analyzing cohorts using different sets may result in an unreliable estimate [28]. We also assume there is no model misspecification when fitting the fully adjusted model. While heterogeneity is inevitable in meta-analysis, CIMBAL does not have a diagnostic test for heterogeneity of fully adjusted estimates from all cohorts. However, we find CIMBAL is not too sensitive to heterogeneity in underlying joint distributions across cohorts; instead it is extremely sensitive to heterogeneity in confounding bias. For instance, age and sex or gender distributions can often be different across studies, and that is not expected to strongly influence CIMBAL as long as the confounding bias is the same across studies. Currently, CIMBAL cannot handle a combination of unadjusted, partially adjusted, and fully adjusted estimates from different cohorts. If many cohorts report partially adjusted estimates with different subsets of confounders, for practical purposes we recommend that investigators choose the most commonly occurring subset of confounder, and regard the corresponding cohorts as ‘complete cohorts’ and the remaining cohorts as those with no confounder information. We suggest that this choice be influenced by not just the number of cohorts reporting a particular set of confounders but also by the sample sizes of such cohorts. Once we have the two groups of cohorts, CIMBAL can be used to impute the ‘fully adjusted’ estimates for the incomplete cohorts as described earlier. We acknowledge this recommendation is sub-optimal: it results in loss of information and statistical efficiency of the meta-analyzed estimate when many cohorts report partially adjusted estimates. Future work will generalize CIMBAL in this aspect.

## Conclusions

Our novel method CIMBAL provides a practical yet valid approach for meta-analyzing independently sampled cohorts with imbalance in measurement of confounders. It is particularly useful when investigators have access to only summary-level data from each cohort. As long as the confounding bias is the same across cohorts, CIMBAL is not too sensitive to heterogeneity in underlying joint distributions of exposure, outcome and confounders. Although we describe CIMBAL in the context of cohort studies, it is also relevant for a meta-analysis of randomized controlled trials.

## Supplementary Information

**Additional file 1** Supplementary materials. The online supplementary materials provide technical proofs, additional figures and tables, and additional data analysis results.

## Data Availability

The NICHD Data and Specimen Hub (DASH) contains the public version of the NCS data (https://dash.nichd.nih.gov/study/228954). The CIMBAL method is implemented in a R program, which is publicly available at https://github.com/RayDebashree/cimbal.

## Abbreviations

BW-for-GA: Birth Weight for Gestational Age
CIMBAL: meta-analysis method for Confounder IMBALance
CKD-PC: Chronic Kidney Disease Prognosis Consortium
CI: Confidence Interval
coRR: confounding Risk Ratio
ECHO: Environmental influences on Child Health Outcomes
GENMETA: GENeralized META-analysis
MSE: Mean Squared Error
NCS: National Children’s Study
SE: Standard Error
