# Supplementary material for: Meta-analysis under imbalance in measurement of confounders in cohort studies using only summary-level data
Source: BMC Med Res Methodol. 2022 May 19;22:143. doi: 10.1186/s12874-022-01614-9 (PMC9118777; doi:10.1186/s12874-022-01614-9)
Supplement: Supplementary file 1 — Additional file 1 Supplementary materials. The online supplementary materials provide technical proofs, additional figures and tables, and additional data analysis results. [file 12874_2022_1614_MOESM1_ESM.pdf]

Supplementary Materials

FOR

Meta-analysis under Imbalance in Measurement of Confounders in Cohort Studies Using Only Summary-level Data

BY

DEBASHREE RAY[a,b,*], ALVARO MUÑOZ[a], MINGYU ZHANG[a], XIUHONG LI[a], NILANJAN CHATTERJEE[b,c], LISA P. JACOBSON[a], BRYAN LAU[a,**]

[a] *Department of Epidemiology, Bloomberg School of Public Health, Johns Hopkins University, USA.*

[b] *Department of Biostatistics, Bloomberg School of Public Health, Johns Hopkins University, USA.*

[c] *Department of Oncology, School of Medicine, Johns Hopkins University, USA.*

[*]dray@jhu.edu; [**]blau1@jhu.edu

Supplementary S1

Empirical demonstration of relations between adjusted and unadjusted estimates from a linear regression setup.

Figure S1: Plot of the difference between adjusted and unadjusted estimates $\hat{\beta}_{\text{unadj}} - \hat{\beta}_{\text{adj}}$ from **linear regression** against sample size. The distribution of this difference is over 2,500 independent replicate datasets for each simulation scenario. For a given parameter setting, this difference in effect estimates stabilizes to a constant as sample size increases, regardless of the strength or direction of the exposure-response association β.
Note: The models used to generate binary exposure X and continuous response Y are respectively $\text{logit}(P(X = 1)) = \eta_0 + \eta_1 C_1 + \eta_2 C_2$ and $Y = \gamma_0 + \gamma_1 C_1 + \gamma_2 C_2 + \beta X + \epsilon$, where confounders $C_1 \sim Bin(1, 0.1)$ and $C_2 \sim Bin(1, 0.6)$, and random error $\epsilon \sim N(0, 1)$. The default parameter settings here assume strong confounder effects: Setting I $\eta_1 = \eta_2 = \gamma_1 = \gamma_2 = 2$; Setting II $\eta_1 = \eta_2 = \gamma_1 = 2, \gamma_2 = -2$; and Setting III $\eta_1 = \eta_2 = 2, \gamma_1 = \gamma_2 = -2$.

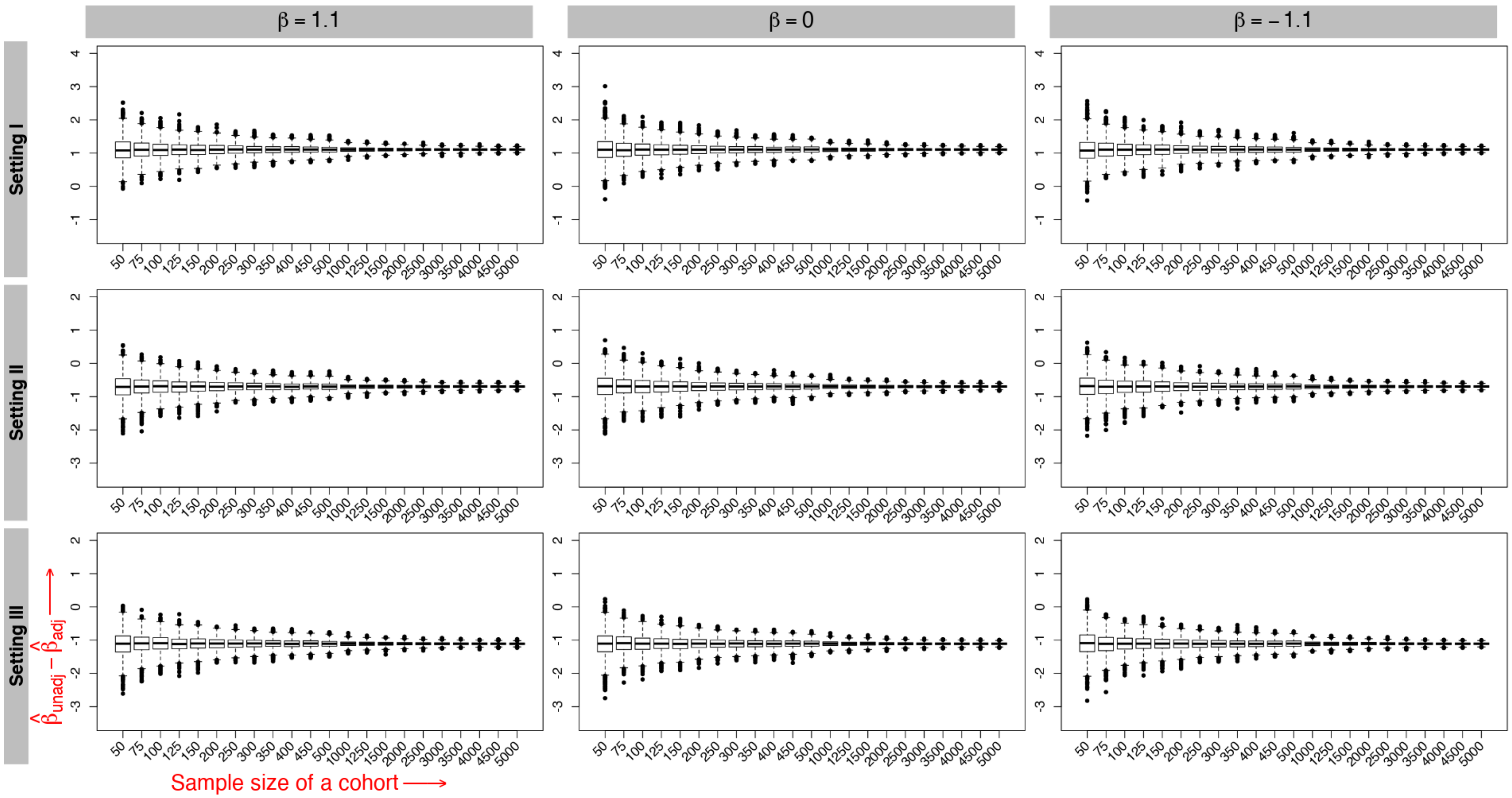

Table S1: Mean of $\hat{\beta}_{\text{unadj}} - \hat{\beta}_{\text{adj}}$ from **linear regression** for increasing sample size. In particular, we generate exposure X and continuous response Y using $X = \eta_0 + \eta_1 C_1 + \eta_2 C_2 + \varepsilon_x$, $\varepsilon_x \sim N(0,1)$ (if continuous) or $\text{logit}(P(X=1)) = \eta_0 + \eta_1 C_1 + \eta_2 C_2$ (if binary) and $Y = \gamma_0 + \gamma_1 C_1 + \gamma_2 C_2 + \beta X + \varepsilon_a$ respectively, where confounders $C_1 \sim Bin(1, 0.1)$ and $C_2 \sim Bin(1, 0.6)$, and random error $\varepsilon_a \sim N(0,1)$. Monte Carlo estimates of the mean is obtained using $10,000$ independent replicate datasets for each simulation scenario. As sample size increases, the difference in effect estimates stabilizes to a constant, regardless of the strength or direction of the exposure-response association β.
Note: The parameter settings here assume intercepts $\eta_0 = 0$ and $\gamma_0 = \log(0.3/0.7) = -0.85$ and the following confounder effects: Setting I (weak) $\eta_1 = \eta_2 = \gamma_1 = \gamma_2 = 0.5$; Setting I (strong) $\eta_1 = \eta_2 = \gamma_1 = \gamma_2 = 2$; Setting II (strong) $\eta_1 = \eta_2 = \gamma_1 = 2, \gamma_2 = -2$; and Setting III (strong) $\eta_1 = \eta_2 = 2, \gamma_1 = \gamma_2 = -2$.

Parameter choices		Sample	**Setting I**		**Setting II**	**Setting III**
distribution	β	size (n)	weak	strong	strong	strong
Y: normal; X: normal; C_1: Bernoulli; C_2: Bernoulli	$\beta = -1.1$	50	0.07647	0.56996	−0.26801	−0.57129
		500	0.07636	0.56958	−0.25912	−0.56893
		2500	0.07623	0.56941	−0.25813	−0.56882
		5000	0.07622	0.56895	−0.25873	−0.56897
	$\beta = 0$	50	0.07665	0.57206	−0.26315	−0.56946
		500	0.0763	0.56883	−0.25981	−0.56926
		2500	0.07621	0.56896	−0.25872	−0.56893
		5000	0.07619	0.56902	−0.25869	−0.56908
	$\beta = 1.1$	50	0.07577	0.57008	−0.26602	−0.57191
		500	0.07603	0.56864	−0.26015	−0.56942
		2500	0.07615	0.56889	−0.25869	−0.56883
		5000	0.07624	0.56907	−0.25862	−0.56906
Y: normal; X: Bernoulli; C_1: Bernoulli; C_2: Bernoulli	$\beta = -1.1$	50	0.07885	1.10329	−0.69597	−1.10421
		500	0.08098	1.10701	−0.69842	−1.10793
		2500	0.08087	1.10715	−0.69827	−1.10738
		5000	0.081	1.10718	−0.69838	−1.10739
	$\beta = 0$	50	0.08352	1.10958	−0.69284	−1.10882
		500	0.08099	1.10858	−0.69905	−1.10903
		2500	0.08104	1.10756	−0.69832	−1.10734
		5000	0.08112	1.10765	−0.69842	−1.10718
	$\beta = 1.1$	50	0.08025	1.10336	−0.70269	−1.11129
		500	0.08113	1.10856	−0.69828	−1.10807
		2500	0.08104	1.10768	−0.69899	−1.10778
		5000	0.08099	1.10704	−0.698	−1.10663

Supplementary S2

Theoretical derivation of relations between adjusted and unadjusted estimates from the linear regression. Consider a continuous response Y, a continuous exposure X and a set of q continuous confounders $\boldsymbol{\mathcal{C}}$. Our interest lies in quantifying the exposure-outcome association. Suppose, at the population-level, the following model holds:

$$Y = \alpha_{\text{adj}} + \beta_{\text{adj}} X + \boldsymbol{\gamma}'\boldsymbol{\mathcal{C}} + \varepsilon_a, \text{ where } \varepsilon_a \sim N(0, \sigma^2_{\text{adj}}) \qquad \text{(True model)}$$
$$X = \eta_0 + \boldsymbol{\eta}'\boldsymbol{\mathcal{C}} + \varepsilon_x, \text{ where } \varepsilon_x \sim N(0, \sigma^2_x)$$
$$\boldsymbol{C} = \boldsymbol{\mu} + \boldsymbol{\varepsilon}_c, \text{ where } \boldsymbol{\varepsilon}_c \sim N(\mathbf{0}, \boldsymbol{\Omega})$$

A cohort, which randomly sampled n individuals from this population and measured Y, X and $\boldsymbol{\mathcal{C}}$ will consider a fully adjusted model to determine exposure-outcome association:

$$Y = \alpha_{\text{adj}} + \beta_{\text{adj}} X + \boldsymbol{\gamma}'\boldsymbol{\mathcal{C}} + \varepsilon_a, \text{ where } \varepsilon_a \sim N(0, \sigma^2_{\text{adj}}) \qquad \text{(Adjusted model)}$$

Here we are making two assumptions: (1) the same probability law $[Y, X, \boldsymbol{\mathcal{C}}]$ (true model) holds for all the populations underlying the different cohorts that employed a random-sampling design; (2) the above fully adjusted model is a correctly specified model for the conditional distribution $[Y|X, \boldsymbol{\mathcal{C}}]$ at the population-level. The cohort may also consider a model without any confounder adjustment:

$$Y = \alpha_{\text{unadj}} + \beta_{\text{unadj}} X + \varepsilon_u, \text{ where } \varepsilon_u \sim N(0, \sigma^2_{\text{unadj}}) \qquad \text{(Unadjusted model)}$$

Note that in the population (true model) all the variables Y, X and $\boldsymbol{\mathcal{C}}$ are considered random. In the sample (adjusted or unadjusted model), Y is treated as random while X and $\boldsymbol{\mathcal{C}}$ are assumed to be fixed. For simplicity of theoretical derivations below, we assume that $\alpha_{\text{unadj}} = 0 = \alpha_{\text{adj}}$, which is satisfied when the variables in the models are centered around their means. For ease of notation, we will use boldfaced lower-case letters to denote vectors or column matrices, boldfaced upper case letters to denote matrices with >1 rows and >1 columns, and the prime symbol ($'$) to denote transpose of a vector/matrix.

Following properties of linear model and some matrix algebra (including block matrix in-

version and Sherman–Morrison–Woodbury matrix identity), the adjusted and the unadjusted estimates of the effect of exposure on outcome (as reported by linear regression functions from standard statistical software) are

$$\begin{aligned}\hat{\beta}_{\text{adj}} &= \left(\boldsymbol{x}'(\boldsymbol{I}-\boldsymbol{P}_{\boldsymbol{\mathcal{C}}})\boldsymbol{x}\right)^{-1}\boldsymbol{x}'(\boldsymbol{I}-\boldsymbol{P}_{\boldsymbol{\mathcal{C}}})\boldsymbol{y}\\ \hat{\beta}_{\text{unadj}} &= \left(\boldsymbol{x}'\boldsymbol{x}\right)^{-1}\boldsymbol{x}'\boldsymbol{y}\end{aligned}$$

where $\boldsymbol{y}$ is the $n\times 1$ vector of outcomes on n individuals, $\boldsymbol{x}$ is the corresponding $n\times 1$ vector of exposure values, $\boldsymbol{C}$ is the corresponding $n\times q$ matrix of confounders (assumed to be of full column rank q), $\boldsymbol{I}$ is an identity matrix of order n, and $\boldsymbol{P}_{\boldsymbol{\mathcal{C}}}=\boldsymbol{C}(\boldsymbol{C}'\boldsymbol{C})^{-1}\boldsymbol{C}'$ is the projection matrix of confounder matrix $\boldsymbol{C}$. As proved below, under the true model,

$$\hat{\beta}_{\text{unadj}}-\hat{\beta}_{\text{adj}} \quad \xrightarrow{P} \quad \frac{\boldsymbol{\eta}'\boldsymbol{\Omega}\boldsymbol{\gamma}}{\sigma_x^2+\boldsymbol{\eta}'\boldsymbol{\Omega}\boldsymbol{\eta}} \text{ as } n\to\infty \tag{S1}$$

where $\xrightarrow{\text{P}}$ denotes convergence in probability. In other words, the difference of the effect estimates are independent of the true exposure-outcome effect size (β_{adj}) and also of the sample size (n) for a large enough cohort.

Derivation of limiting form of the difference of effect estimates from the linear regression.

Proof. Using properties of linear model and matrix algebra, we have

$$\begin{aligned}\mathrm{E}_{[Y|X,\boldsymbol{\mathcal{C}}]}\left(\hat{\beta}_{\text{adj}}\right) &= \left(\boldsymbol{x}'(\boldsymbol{I}-\boldsymbol{P}_{\boldsymbol{\mathcal{C}}})\boldsymbol{x}\right)^{-1}\boldsymbol{x}'(\boldsymbol{I}-\boldsymbol{P}_{\boldsymbol{\mathcal{C}}})\,\mathrm{E}_{[Y|X,\boldsymbol{\mathcal{C}}]}(\boldsymbol{y})\\ &= \left(\boldsymbol{x}'(\boldsymbol{I}-\boldsymbol{P}_{\boldsymbol{\mathcal{C}}})\boldsymbol{x}\right)^{-1}\boldsymbol{x}'(\boldsymbol{I}-\boldsymbol{P}_{\boldsymbol{\mathcal{C}}})[\boldsymbol{x}\beta_{\text{adj}}+\boldsymbol{C}\boldsymbol{\gamma}]\\ &= \beta_{\text{adj}},\ \text{ since } (\boldsymbol{I}-\boldsymbol{P}_{\boldsymbol{\mathcal{C}}})\boldsymbol{C}=\mathrm{O}\\ \text{and, }\ \mathrm{E}_{[Y|X,\boldsymbol{\mathcal{C}}]}\left(\hat{\beta}_{\text{unadj}}\right) &= \left(\boldsymbol{x}'\boldsymbol{x}\right)^{-1}\boldsymbol{x}'\,\mathrm{E}_{[Y|X]}(\boldsymbol{y})\end{aligned}$$

Under the true model, the distribution of Y given X can be obtained as

$$[Y|X]=\int_{\boldsymbol{\mathcal{C}}}[Y,\boldsymbol{\mathcal{C}}|X]=\int_{\boldsymbol{\mathcal{C}}}[Y|X,\boldsymbol{\mathcal{C}}]\ [\boldsymbol{\mathcal{C}}|X]$$

While we know the distribution $[Y|X, \boldsymbol{\mathcal{C}}]$, we need to obtain distribution $[\boldsymbol{\mathcal{C}}|X]$ from the joint distribution $[X, \boldsymbol{\mathcal{C}}]$. For i-th individual, $x_i = \boldsymbol{\eta}'\boldsymbol{\mathcal{C}}_i + \varepsilon_{x,i}$, where $\boldsymbol{\mathcal{C}}_i \overset{iid}{\sim} N_q(\mathbf{0}, \boldsymbol{\Omega})$ and $\varepsilon_{x,i} \overset{iid}{\sim} N(0, \sigma_x^2)\ \forall\ i = 1, 2, ..., n$. This gives $\mathrm{E}(x_i) = 0$, $\mathrm{Var}(x_i) = \mathrm{Var}(\boldsymbol{\eta}'\boldsymbol{\mathcal{C}}_i) + \mathrm{Var}(\varepsilon_{x,i}) = \boldsymbol{\eta}'\boldsymbol{\Omega}\boldsymbol{\eta} + \sigma_x^2$ and $\mathrm{Cov}(\boldsymbol{\mathcal{C}}_i, x_i) = \mathrm{Cov}(\boldsymbol{\mathcal{C}}_i, \boldsymbol{\eta}'\boldsymbol{\mathcal{C}}_i) = \boldsymbol{\Omega}\boldsymbol{\eta}$. Thus, the marginal distribution $[X]$ is $x_i \overset{iid}{\sim} N(0, \sigma_x^2 + \boldsymbol{\eta}'\boldsymbol{\Omega}\boldsymbol{\eta})\ \forall\ i$ and the joint distribution $[X, \boldsymbol{\mathcal{C}}]$ is

$$\begin{pmatrix} \boldsymbol{\mathcal{C}}_i \\ x_i \end{pmatrix} \overset{iid}{\sim} N_{q+1}\left(\begin{bmatrix} \mathbf{0} \\ 0 \end{bmatrix}, \begin{bmatrix} \boldsymbol{\Omega} & \boldsymbol{\Omega}\boldsymbol{\eta} \\ \boldsymbol{\eta}'\boldsymbol{\Omega} & \sigma_x^2 + \boldsymbol{\eta}'\boldsymbol{\Omega}\boldsymbol{\eta} \end{bmatrix} \right)\ \forall\ i$$

Using conditional distribution property of multivariate normal distribution, we get $[\boldsymbol{\mathcal{C}}|X]$:

$$\boldsymbol{\mathcal{C}}_i \Big| x_i \overset{ind}{\sim} N_q\left(\frac{\boldsymbol{\Omega}\boldsymbol{\eta}}{\sigma_x^2 + \boldsymbol{\eta}'\boldsymbol{\Omega}\boldsymbol{\eta}} x_i\ ,\ \boldsymbol{\Omega} - \frac{\boldsymbol{\Omega}\boldsymbol{\eta}\boldsymbol{\eta}'\boldsymbol{\Omega}}{\sigma_x^2 + \boldsymbol{\eta}'\boldsymbol{\Omega}\boldsymbol{\eta}} \right)\ \forall\ i \tag{S2}$$

For the i-th individual, given x_i, we have $y_i = \beta_{\text{adj}} x_i + \boldsymbol{\gamma}'\boldsymbol{\mathcal{C}}_i + \varepsilon_{a,i}$, where $\boldsymbol{\mathcal{C}}_i$ has the distribution from equation (S2) and $\varepsilon_{a,i} \overset{iid}{\sim} N(0, \sigma_{\text{adj}}^2)\ \forall\ i$. The required distribution $[Y|X]$ is then

$$Y_i \Big| x_i \overset{ind}{\sim} N\left(\left[\beta_{\text{adj}} + \frac{\boldsymbol{\gamma}'\boldsymbol{\Omega}\boldsymbol{\eta}}{\sigma_x^2 + \boldsymbol{\eta}'\boldsymbol{\Omega}\boldsymbol{\eta}} \right] x_i\ ,\ \sigma_{\text{adj}}^2 + \boldsymbol{\gamma}' \left[\boldsymbol{\Omega} - \frac{\boldsymbol{\Omega}\boldsymbol{\eta}\boldsymbol{\eta}'\boldsymbol{\Omega}}{\sigma_x^2 + \boldsymbol{\eta}'\boldsymbol{\Omega}\boldsymbol{\eta}} \right] \boldsymbol{\gamma} \right)$$

Thus, in the true underlying population,

$$\mathrm{E}_{\text{true}}\left(\hat{\beta}_{\text{adj}}\right) = \mathrm{E}_{[Y,X,\boldsymbol{\mathcal{C}}]}\left(\hat{\beta}_{\text{adj}}\right) = \mathrm{E}_{[X,\boldsymbol{\mathcal{C}}]}\left(\mathrm{E}_{[Y|X,\boldsymbol{\mathcal{C}}]}\left(\hat{\beta}_{\text{adj}}\right)\right) = \beta_{\text{adj}} \tag{S3}$$

$$\begin{aligned} \text{and,}\ \mathrm{E}_{\text{true}}\left(\hat{\beta}_{\text{unadj}}\right) &= \mathrm{E}_{[Y,X,\boldsymbol{\mathcal{C}}]}\left(\hat{\beta}_{\text{unadj}}\right) = \mathrm{E}_{[X,\boldsymbol{\mathcal{C}}]}\left(\mathrm{E}_{[Y|X,\boldsymbol{\mathcal{C}}]}\left(\hat{\beta}_{\text{unadj}}\right)\right) \\ &= \mathrm{E}_{[X,\boldsymbol{\mathcal{C}}]}\left((\boldsymbol{x}'\boldsymbol{x})^{-1} \boldsymbol{x}'\, \mathrm{E}_{[Y|X]}(\boldsymbol{y}) \right) \\ &= \beta_{\text{adj}} + \frac{\boldsymbol{\gamma}'\boldsymbol{\Omega}\boldsymbol{\eta}}{\sigma_x^2 + \boldsymbol{\eta}'\boldsymbol{\Omega}\boldsymbol{\eta}} \end{aligned} \tag{S4}$$

Side note: in the econometrics literature, 'omitted variable bias' refers to the term

$$\mathrm{E}_{[Y|X,\boldsymbol{\mathcal{C}}]}\left(\hat{\beta}_{\text{unadj}}\right) - \beta_{\text{adj}} = (\boldsymbol{x}'\boldsymbol{x})^{-1} \boldsymbol{x}'\, \mathrm{E}_{[Y|X]}(\boldsymbol{y}) - \beta_{\text{adj}} = \frac{\boldsymbol{\gamma}'\boldsymbol{\Omega}\boldsymbol{\eta}}{\sigma_x^2 + \boldsymbol{\eta}'\boldsymbol{\Omega}\boldsymbol{\eta}}$$

and it is 0 when $\boldsymbol{\gamma} = \mathbf{0}$ or when $\mathrm{Cov}(\boldsymbol{\mathcal{C}}, X) = \boldsymbol{\Omega}\boldsymbol{\eta} = \mathbf{0}$. Finally, using weak law of large numbers

and properties of convergence in probability,

$$\hat{\beta}_{\text{unadj}} - \hat{\beta}_{\text{adj}} \xrightarrow{P} \mathrm{E}_{\text{true}}\left(\hat{\beta}_{\text{unadj}}\right) - \mathrm{E}_{\text{true}}\left(\hat{\beta}_{\text{adj}}\right) = \frac{\boldsymbol{\gamma}'\boldsymbol{\Omega}\boldsymbol{\eta}}{\sigma_x^2 + \boldsymbol{\eta}'\boldsymbol{\Omega}\boldsymbol{\eta}} \text{ as } n \to \infty$$

■

Empirical proof of the limiting forms when the underlying data generating mechanism is the same as the assumed true model. Refer Figure S2 and Table S2.

Proof of non-negative covariance between adjusted and unadjusted effect estimates from the linear regression.

Proof. The form for covariance in the true underlying population is

$$\mathrm{Cov}_{\text{true}}(\hat{\beta}_{\text{unadj}}, \hat{\beta}_{\text{adj}}) = \mathrm{E}_{[Y,X,\boldsymbol{\mathcal{C}}]}(\hat{\beta}_{\text{unadj}}\hat{\beta}_{\text{adj}}) - \mathrm{E}_{[Y,X,\boldsymbol{\mathcal{C}}]}(\hat{\beta}_{\text{unadj}})\,\mathrm{E}_{[Y,X,\boldsymbol{\mathcal{C}}]}(\hat{\beta}_{\text{adj}})$$

where the individual expectation terms have been obtained in equations (S3) and (S4). Now,

$$\begin{aligned}
&\mathrm{E}_{[Y,X,\boldsymbol{\mathcal{C}}]}(\hat{\beta}_{\text{unadj}}\,\hat{\beta}_{\text{adj}}) \\
&= \mathrm{E}_{[X,\boldsymbol{\mathcal{C}}]}\left\{\mathrm{E}_{[Y|X,\boldsymbol{\mathcal{C}}]}\left(\frac{\boldsymbol{x}'(\boldsymbol{I}-\boldsymbol{P}_{\boldsymbol{\mathcal{C}}})\boldsymbol{y}}{\boldsymbol{x}'(\boldsymbol{I}-\boldsymbol{P}_{\boldsymbol{\mathcal{C}}})\boldsymbol{x}} \times \frac{\boldsymbol{x}'\boldsymbol{y}}{\boldsymbol{x}'\boldsymbol{x}}\right)\right\} \\
&= \mathrm{E}_{[X,\boldsymbol{\mathcal{C}}]}\left\{\frac{1}{\boldsymbol{x}'(\boldsymbol{I}-\boldsymbol{P}_{\boldsymbol{\mathcal{C}}})\boldsymbol{x}\;\boldsymbol{x}'\boldsymbol{x}}\;\mathrm{E}_{[Y|X,\boldsymbol{\mathcal{C}}]}\left(\boldsymbol{y}'\,(\boldsymbol{I}-\boldsymbol{P}_{\boldsymbol{\mathcal{C}}})\boldsymbol{x}\boldsymbol{x}'\,\boldsymbol{y}\right)\right\} \\
&= \mathrm{E}_{[X,\boldsymbol{\mathcal{C}}]}\left\{\frac{1}{\boldsymbol{x}'(\boldsymbol{I}-\boldsymbol{P}_{\boldsymbol{\mathcal{C}}})\boldsymbol{x}\;\boldsymbol{x}'\boldsymbol{x}}\left[\mathrm{tr}\left((\boldsymbol{I}-\boldsymbol{P}_{\boldsymbol{\mathcal{C}}})\boldsymbol{x}\boldsymbol{x}'\,\sigma^2_{\text{adj}}\boldsymbol{I}_n\right) + \beta_{\text{adj}}\boldsymbol{x}'(\boldsymbol{I}-\boldsymbol{P}_{\boldsymbol{\mathcal{C}}})\boldsymbol{x}\left(\beta_{\text{adj}}\boldsymbol{x}'\boldsymbol{x} + \boldsymbol{x}'\boldsymbol{C}\boldsymbol{\gamma}\right)\right]\right\} \\
&\qquad \text{(using the form for expectation of a quadratic form)} \\
&= \mathrm{E}_{[X,\boldsymbol{\mathcal{C}}]}\left\{\frac{1}{\boldsymbol{x}'(\boldsymbol{I}-\boldsymbol{P}_{\boldsymbol{\mathcal{C}}})\boldsymbol{x}\;\boldsymbol{x}'\boldsymbol{x}}\left[\sigma^2_{\text{adj}}\;\boldsymbol{x}'(\boldsymbol{I}-\boldsymbol{P}_{\boldsymbol{\mathcal{C}}})\boldsymbol{x} + \beta_{\text{adj}}\boldsymbol{x}'(\boldsymbol{I}-\boldsymbol{P}_{\boldsymbol{\mathcal{C}}})\boldsymbol{x}\left(\beta_{\text{adj}}\boldsymbol{x}'\boldsymbol{x} + \boldsymbol{x}'\boldsymbol{C}\boldsymbol{\gamma}\right)\right]\right\} \\
&= \sigma^2_{\text{adj}}\;\mathrm{E}_{[X,\boldsymbol{\mathcal{C}}]}\left(\frac{1}{\boldsymbol{x}'\boldsymbol{x}}\right) + \beta^2_{\text{adj}} + \beta_{\text{adj}}\;\mathrm{E}_{[X,\boldsymbol{\mathcal{C}}]}\left(\frac{\boldsymbol{x}'\boldsymbol{C}\boldsymbol{\gamma}}{\boldsymbol{x}'\boldsymbol{x}}\right)
\end{aligned}$$

which leads to

$$\begin{aligned}
\mathrm{Cov}_{\text{true}}(\hat{\beta}_{\text{unadj}}, \hat{\beta}_{\text{adj}}) &= \left[\sigma^2_{\text{adj}}\;\mathrm{E}_{[X,\boldsymbol{\mathcal{C}}]}\left(\frac{1}{\boldsymbol{x}'\boldsymbol{x}}\right) + \beta^2_{\text{adj}} + \beta_{\text{adj}}\;\mathrm{E}_{[X,\boldsymbol{\mathcal{C}}]}\left(\frac{\boldsymbol{x}'\boldsymbol{C}\boldsymbol{\gamma}}{\boldsymbol{x}'\boldsymbol{x}}\right)\right] - \left[\beta_{\text{adj}} + \frac{\boldsymbol{\gamma}'\boldsymbol{\Omega}\boldsymbol{\eta}}{\sigma_x^2 + \boldsymbol{\eta}'\boldsymbol{\Omega}\boldsymbol{\eta}}\right]\beta_{\text{adj}} \\
&= \sigma^2_{\text{adj}}\;\mathrm{E}_{[X,\boldsymbol{\mathcal{C}}]}\left(\frac{1}{\boldsymbol{x}'\boldsymbol{x}}\right) + \beta_{\text{adj}}\left[\mathrm{E}_{[X,\boldsymbol{\mathcal{C}}]}\left(\frac{\boldsymbol{x}'\boldsymbol{C}\boldsymbol{\gamma}}{\boldsymbol{x}'\boldsymbol{x}}\right) - \frac{\boldsymbol{\gamma}'\boldsymbol{\Omega}\boldsymbol{\eta}}{\sigma_x^2 + \boldsymbol{\eta}'\boldsymbol{\Omega}\boldsymbol{\eta}}\right]
\end{aligned}$$

To obtain $\mathrm{E}_{[X,\mathcal{C}]}\left(\frac{\boldsymbol{x}'\boldsymbol{C}\boldsymbol{\gamma}}{\boldsymbol{x}'\boldsymbol{x}}\right) = \mathrm{E}_{[X]}\left(\frac{1}{\boldsymbol{x}'\boldsymbol{x}}\ \mathrm{E}_{[\mathcal{C}|X]}(\boldsymbol{x}'\boldsymbol{C}\boldsymbol{\gamma})\right)$, note that we can write $\boldsymbol{C}\boldsymbol{\gamma} = \begin{pmatrix} \mathcal{C}_1'\gamma \\ \vdots \\ \mathcal{C}_n'\gamma \end{pmatrix}$ where $\boldsymbol{\gamma}'\mathcal{C}_i\big|x_i \overset{ind}{\sim} N\left(\frac{\boldsymbol{\gamma}'\boldsymbol{\Omega}\boldsymbol{\eta}}{\sigma_x^2+\boldsymbol{\eta}'\boldsymbol{\Omega}\boldsymbol{\eta}}x_i\ ,\ \boldsymbol{\gamma}'\left\{\boldsymbol{\Omega} - \frac{\boldsymbol{\Omega}\boldsymbol{\eta}\boldsymbol{\eta}'\boldsymbol{\Omega}}{\sigma_x^2+\boldsymbol{\eta}'\boldsymbol{\Omega}\boldsymbol{\eta}}\right\}\boldsymbol{\gamma}\right)\ \forall\ i = 1, 2, ..., n$ using the conditional distribution $[\mathcal{C}|X]$ derived before. Consequently, $\boldsymbol{C}\boldsymbol{\gamma} \sim N_q\left(\frac{\boldsymbol{\gamma}'\boldsymbol{\Omega}\boldsymbol{\eta}}{\sigma_x^2+\boldsymbol{\eta}'\boldsymbol{\Omega}\boldsymbol{\eta}}\boldsymbol{x}\ ,\ \boldsymbol{\gamma}'\left\{\boldsymbol{\Omega} - \frac{\boldsymbol{\Omega}\boldsymbol{\eta}\boldsymbol{\eta}'\boldsymbol{\Omega}}{\sigma_x^2+\boldsymbol{\eta}'\boldsymbol{\Omega}\boldsymbol{\eta}}\right\}\boldsymbol{\gamma}\boldsymbol{I}_n\right)$ and $\mathrm{E}_{[\mathcal{C}|X]}(\boldsymbol{x}'\boldsymbol{C}\boldsymbol{\gamma}) = \frac{\boldsymbol{\gamma}'\boldsymbol{\Omega}\boldsymbol{\eta}}{\sigma_x^2+\boldsymbol{\eta}'\boldsymbol{\Omega}\boldsymbol{\eta}}\boldsymbol{x}'\boldsymbol{x}$. Therefore,

$$\mathrm{Cov}_{\mathrm{true}}(\hat{\beta}_{\mathrm{unadj}}, \hat{\beta}_{\mathrm{adj}}) = \sigma^2_{\mathrm{adj}}\ \mathrm{E}_{[X,\mathcal{C}]}\left(\frac{1}{\boldsymbol{x}'\boldsymbol{x}}\right) \geq 0$$

since $\boldsymbol{x}'\boldsymbol{x}$ is a positive random variable (note, given $\mathcal{C}$, $\boldsymbol{x}'\boldsymbol{x}$ has a scaled non-central χ^2 distribution). ■

Figure S2: Plot of the difference between adjusted and unadjusted estimates $\hat{\beta}_{\text{unadj}} - \hat{\beta}_{\text{adj}}$ from **linear regression** against sample size when **the data generating mechanism is the same as the true model assumed in above theoretical proofs**. In particular, we generate continuous exposure X and continuous response Y using $X = \eta_0 + \eta_1 C_1 + \eta_2 C_2 + \varepsilon_x$ and $Y = \gamma_0 + \gamma_1 C_1 + \gamma_2 C_2 + \beta X + \varepsilon_a$ respectively, where confounders C_1 and C_2 are generated from a bivariate normal distribution with means 0, variances 1 and correlation 0.7; and random errors $\varepsilon_x \sim N(0,1)$ and $\varepsilon_a \sim N(0,1)$. The distribution of this difference $\hat{\beta}_{\text{unadj}} - \hat{\beta}_{\text{adj}}$ is over $2,500$ independent replicate datasets for each simulation scenario. For a given parameter setting, this difference in effect estimates stabilizes to a constant as sample size increases, regardless of the strength or direction of the exposure-response association β.
Note: The default parameter settings here assume zero intercepts ($\eta_0 = 0, \gamma_0 = 0$) and strong confounder effects: Setting I $\eta_1 = \eta_2 = \gamma_1 = \gamma_2 = 2$; Setting II $\eta_1 = \eta_2 = \gamma_1 = 2, \gamma_2 = -2$; and Setting III $\eta_1 = \eta_2 = 2, \gamma_1 = \gamma_2 = -2$. The dashed horizontal line corresponds to the theoretical limiting value $\frac{\boldsymbol{\eta}'\boldsymbol{\Omega}\boldsymbol{\gamma}}{\sigma_x^2 + \boldsymbol{\eta}'\boldsymbol{\Omega}\boldsymbol{\eta}}$, which equals 0.93 (Setting I), 0 (Setting II) or -0.93 (Setting III) respectively.

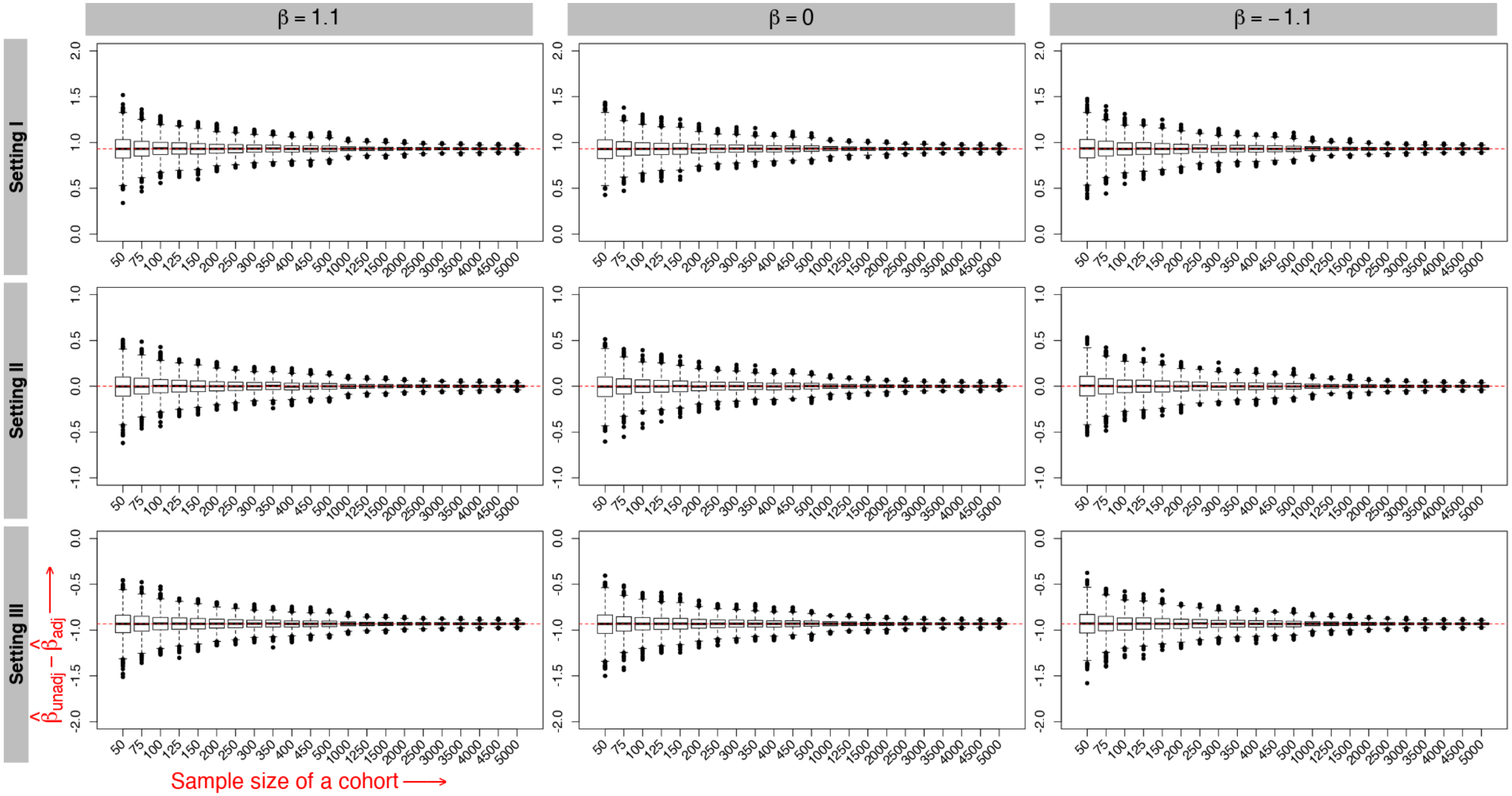

Table S2: Mean squared deviation of $\hat{\beta}_{\text{unadj}} - \hat{\beta}_{\text{adj}}$ from the theoretical limit we obtained for **linear regression** with the **data generating mechanism same as the true model assumed in above theoretical proofs**. In particular, we generate continuous exposure X and continuous response Y using $X = \eta_0 + \eta_1 C_1 + \eta_2 C_2 + \varepsilon_x$ and $Y = \gamma_0 + \gamma_1 C_1 + \gamma_2 C_2 + \beta X + \varepsilon_a$ respectively, where confounders C_1 and C_2 are generated from a bivariate normal distribution with means 0, variances 1 and correlation 0.7; and random errors $\varepsilon_x \sim N(0, \sigma_x^2)$ and $\varepsilon_a \sim N(0, \sigma_{\text{adj}}^2)$. Monte Carlo estimates of the mean squared deviation is obtained using 10,000 independent replicate datasets for each simulation scenario. For a given parameter setting, the ideal cell value is 0. As sample size increases, we observe nearly 0 deviation, indicating the difference in effect estimates stabilizes to the theoretical limit $\frac{\boldsymbol{\eta}'\boldsymbol{\Omega}\boldsymbol{\gamma}}{\sigma_x^2 + \boldsymbol{\eta}'\boldsymbol{\Omega}\boldsymbol{\eta}}$ (a constant), regardless of the strength or direction of the exposure-response association β.
Note: The parameter settings here assume zero intercepts ($\eta_0 = 0, \gamma_0 = 0$) and the following confounder effects: Setting I (weak) $\eta_1 = \eta_2 = \gamma_1 = \gamma_2 = 0.5$; Setting I (strong) $\eta_1 = \eta_2 = \gamma_1 = \gamma_2 = 2$; Setting II (strong) $\eta_1 = \eta_2 = \gamma_1 = 2, \gamma_2 = -2$; and Setting III (strong) $\eta_1 = \eta_2 = 2, \gamma_1 = \gamma_2 = -2$.

Parameter choices		Sample	**Setting I**		**Setting II**	**Setting III**
$(\sigma_x, \sigma_{\text{adj}})$	β	size (n)	weak	strong	strong	strong
$\sigma_x = 1$, $\sigma_{\text{adj}} = 1$	$\beta = -1.1$	50	0.0161	0.02268	0.02481	0.02252
		500	0.00141	0.00198	0.00219	0.002
		2500	0.00029	0.0004	0.00044	0.0004
		5000	0.00014	0.0002	0.00022	0.0002
	$\beta = 0$	50	0.01622	0.02265	0.0243	0.02241
		500	0.00142	0.00199	0.0022	0.00201
		2500	0.00029	0.00041	0.00045	0.00041
		5000	0.00015	0.0002	0.00022	0.0002
	$\beta = 1.1$	50	0.01629	0.02261	0.02495	0.02247
		500	0.0014	0.00198	0.0022	0.00199
		2500	0.00028	0.0004	0.00044	0.0004
		5000	0.00014	0.0002	0.00022	0.0002
$\sigma_x = 3$, $\sigma_{\text{adj}} = 2$	$\beta = -1.1$	50	0.00444	0.01129	0.00851	0.01137
		500	0.00033	0.001	0.00074	0.00102
		2500	0.00007	0.0002	0.00015	0.0002
		5000	0.00003	0.0001	0.00007	0.0001
	$\beta = 0$	50	0.00465	0.0113	0.00832	0.0113
		500	0.00034	0.00101	0.00076	0.00103
		2500	0.00006	0.00021	0.00015	0.00021
		5000	0.00003	0.0001	0.00008	0.0001
	$\beta = 1.1$	50	0.00459	0.01137	0.00862	0.0114
		500	0.00034	0.001	0.00075	0.00104
		2500	0.00007	0.0002	0.00015	0.0002
		5000	0.00003	0.0001	0.00008	0.0001
$\sigma_x = 0.5$, $\sigma_{\text{adj}} = 2$	$\beta = -1.1$	50	0.28868	0.35746	0.36087	0.35689
		500	0.02503	0.03146	0.03176	0.03149
		2500	0.00505	0.00635	0.00641	0.00636
		5000	0.00249	0.00315	0.00318	0.00314
	$\beta = 0$	50	0.28795	0.3571	0.35861	0.35653
		500	0.02528	0.03164	0.03194	0.03168
		2500	0.00512	0.00645	0.00651	0.00646
		5000	0.00261	0.00321	0.00323	0.0032
	$\beta = 1.1$	50	0.28764	0.356	0.36029	0.35561
		500	0.02486	0.0314	0.03173	0.03142
		2500	0.00506	0.00638	0.00645	0.00638
		5000	0.00253	0.00317	0.0032	0.00318

Supplementary S3

Empirical demonstration of relations between adjusted and unadjusted estimates from a logistic regression setup.

Figure S3: Plot of the difference between adjusted and unadjusted estimates $\hat{\beta}_{\text{unadj}} - \hat{\beta}_{\text{adj}} = \log(\hat{\text{OR}}_{\text{unadj}}) - \log(\hat{\text{OR}}_{\text{adj}})$ from **logistic regression** against sample size. The distribution of this difference is over 2,500 independent replicate datasets for each simulation scenario. For a given parameter setting, this difference in effect estimates stabilizes to a constant as sample size increases, regardless of the strength or direction of the exposure-outcome association β.
Note: The models used to generate binary exposure X and binary outcome Y are respectively $\text{logit}(X) = \eta_0 + \eta_1 C_1 + \eta_2 C_2$ and $\text{logit}(Y) = \gamma_0 + \gamma_1 C_1 + \gamma_2 C_2 + \beta X$, where confounders $C_1 \sim Bin(1, 0.1)$ and $C_2 \sim Bin(1, 0.6)$. The default parameter settings here assume strong confounder effects: Setting I $\eta_1 = \eta_2 = \gamma_1 = \gamma_2 = 2$; Setting II $\eta_1 = \eta_2 = \gamma_1 = 2, \gamma_2 = -2$; and Setting III $\eta_1 = \eta_2 = 2, \gamma_1 = \gamma_2 = -2$.

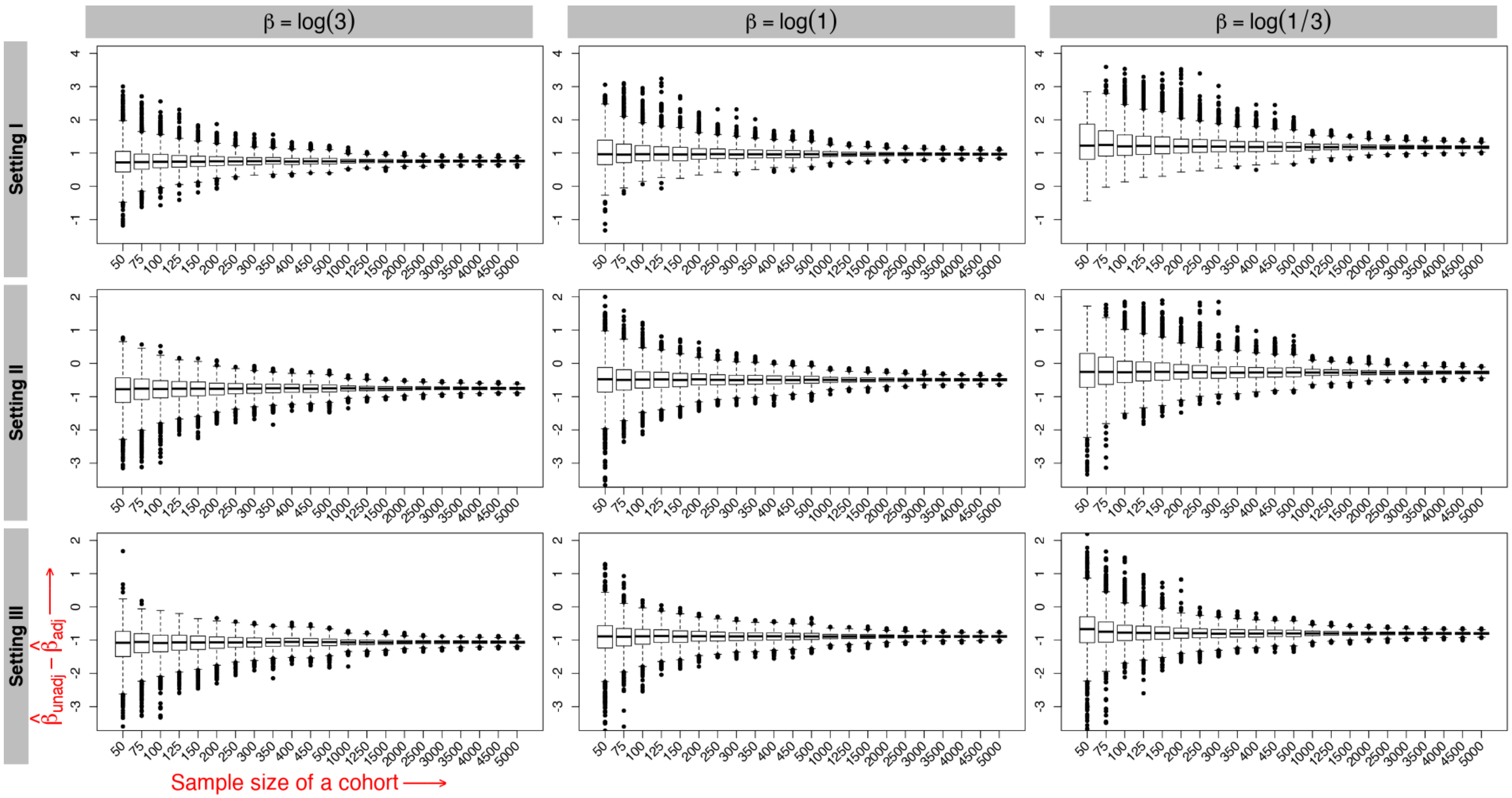

Table S3: Mean of $\hat{\beta}_{\text{unadj}} - \hat{\beta}_{\text{adj}}$ from **logistic regression** for increasing sample size. In particular, we generate exposure X and binary response Y using $X = \eta_0 + \eta_1 C_1 + \eta_2 C_2 + \varepsilon_x$, $\varepsilon_x \sim N(0,1)$ (if continuous) or $\text{logit}(P(X=1)) = \eta_0 + \eta_1 C_1 + \eta_2 C_2$ (if binary) and $\text{logit}(P(Y=1)) = \gamma_0 + \gamma_1 C_1 + \gamma_2 C_2 + \beta X$ respectively, where confounders $C_1 \sim Bin(1, 0.1)$ and $C_2 \sim Bin(1, 0.6)$. Monte Carlo estimates of the mean is obtained using $10,000$ independent replicate datasets for each simulation scenario. For a given exposure-response association β, the difference in effect estimates stabilizes to a constant as sample size increases.
Note: The parameter settings here assume intercepts $\eta_0 = 0$ and $\gamma_0 = \log(0.3/0.7) = -0.85$ and the following confounder effects: Setting I (weak) $\eta_1 = \eta_2 = \gamma_1 = \gamma_2 = 0.5$; Setting I (strong) $\eta_1 = \eta_2 = \gamma_1 = \gamma_2 = 2$; Setting II (strong) $\eta_1 = \eta_2 = \gamma_1 = 2, \gamma_2 = -2$; and Setting III (strong) $\eta_1 = \eta_2 = 2, \gamma_1 = \gamma_2 = -2$.

Parameter choices		Sample	**Setting I**		**Setting II**	**Setting III**
distribution	β	size (n)	weak	strong	strong	strong
Y: Bernoulli; X: normal; C_1: Bernoulli; C_2: Bernoulli	$\beta = \log\frac{1}{3}$	50	0.1709	0.76841	−0.00256	−0.2265
		500	0.09741	0.63152	−0.16071	−0.38805
		2500	0.09232	0.62289	−0.16467	−0.39216
		5000	0.09151	0.62159	−0.16647	−0.39293
	$\beta = \log 1$	50	0.08151	0.58704	−0.21792	−0.52686
		500	0.07648	0.54461	−0.20021	−0.51571
		2500	0.07532	0.5413	−0.19954	−0.51154
		5000	0.07544	0.54085	−0.19908	−0.5109
	$\beta = \log 3$	50	−0.00013	0.47773	−0.53895	−0.7544
		500	0.05406	0.53742	−0.44531	−0.63836
		2500	0.05794	0.53586	−0.43778	−0.62874
		5000	0.05841	0.53722	−0.43722	−0.62797
Y: Bernoulli; X: Bernoulli; C_1: Bernoulli; C_2: Bernoulli	$\beta = \log\frac{1}{3}$	50	0.22026	2.3338	0.87365	−0.51835
		500	0.10349	1.20115	−0.26778	−0.79768
		2500	0.09842	1.18244	−0.27437	−0.79766
		5000	0.09809	1.18002	−0.27696	−0.79764
	$\beta = \log 1$	50	0.09168	1.1915	−0.41034	−0.84504
		500	0.08077	0.98065	−0.49172	−0.89175
		2500	0.08001	0.97122	−0.49224	−0.88865
		5000	0.08	0.96917	−0.49174	−0.88799
	$\beta = \log 3$	50	−0.00034	0.73776	−0.95603	−1.22719
		500	0.05497	0.76418	−0.75791	−1.06999
		2500	0.05779	0.76125	−0.7513	−1.06165
		5000	0.0585	0.76231	−0.7507	−1.06135

Figure S4: Plot of estimated covariance between unadjusted and adjusted estimates against the number of cohorts with complete confounder information used to estimate the covariance. Sample size for each cohort is 150. The distribution of estimated Cov $\left(\hat{\beta}_{\text{unadj}}, \hat{\beta}_{\text{adj}}\right)$ is over 2,500 independent replicate datasets for each simulation scenario. The horizontal dashed line correspond to the theoretical lower limit of this covariance for the linear regression case. The number of complete cohorts needed to estimate this covariance depends on the strengths and directions of the confounder effects as well as the exposure-outcome association.

Note: The models used to generate binary exposure X and binary outcome Y are respectively $\text{logit}(X) = \eta_0 + \eta_1 C_1 + \eta_2 C_2$ and $\text{logit}(Y) = \gamma_0 + \gamma_1 C_1 + \gamma_2 C_2 + \beta X$, where confounders $C_1 \sim Bin(1, 0.1)$ and $C_2 \sim Bin(1, 0.6)$. The default parameter settings here assume strong confounder effects: Setting I $\eta_1 = \eta_2 = \gamma_1 = \gamma_2 = 2$; Setting II $\eta_1 = \eta_2 = \gamma_1 = 2, \gamma_2 = -2$; and Setting III $\eta_1 = \eta_2 = 2, \gamma_1 = \gamma_2 = -2$. Only Setting I "weak" assumes weak confounder effects: $\eta_1 = \eta_2 = \gamma_1 = \gamma_2 = 0.5$.

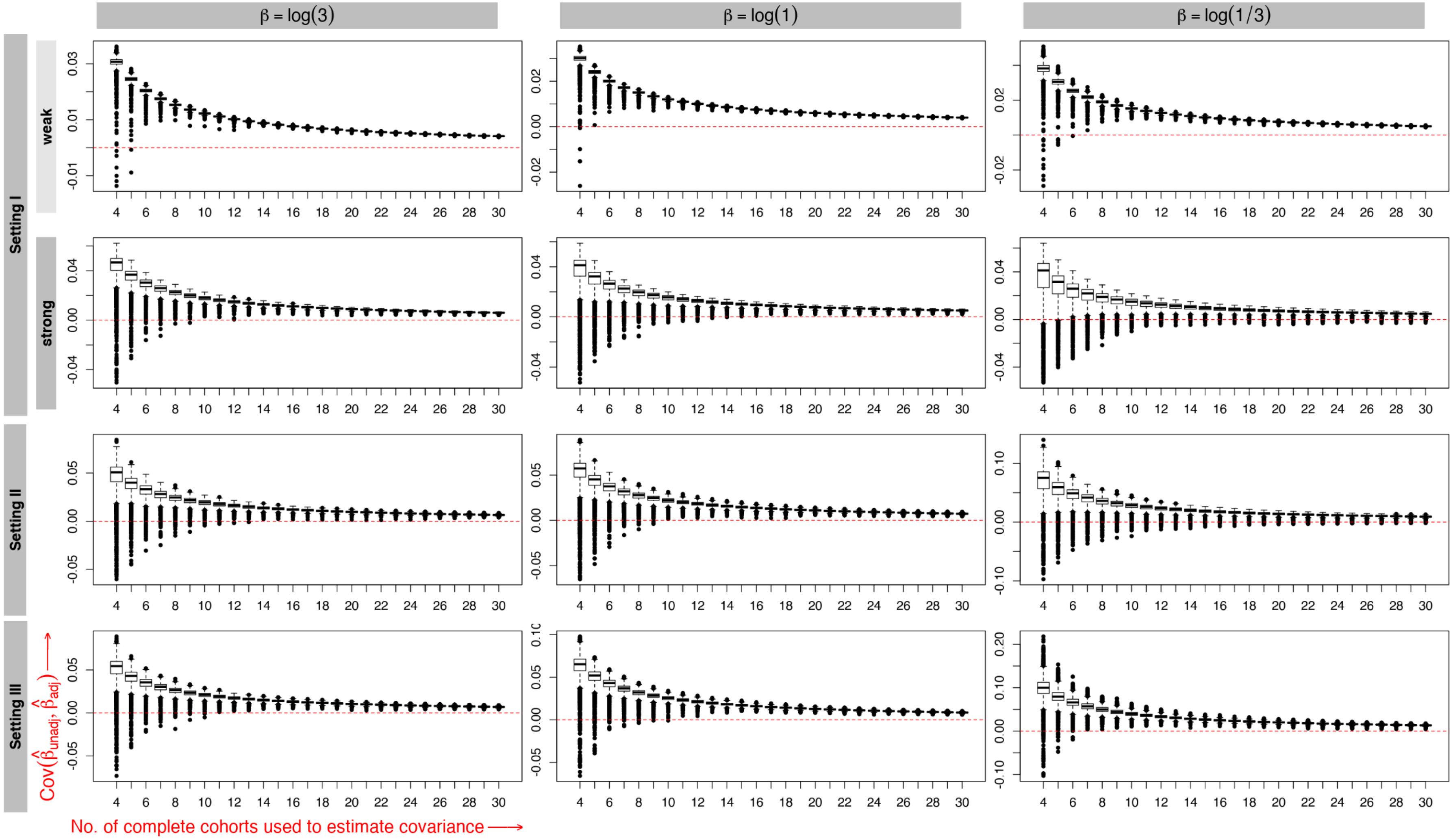

Figure S5: Plot of Pearson's correlation coefficient between unadjusted and adjusted effect estimates against the number of cohorts with complete confounder information used to estimate the covariance in CIMBAL. Sample size for each cohort is 150. The distribution of estimated $\left(\hat{\beta}_{\text{unadj}}, \hat{\beta}_{\text{adj}}\right)$ is over $2,500$ independent replicate datasets for each simulation scenario. The number of complete cohorts needed to estimate this covariance depends on the strengths and directions of the confounder effects as well as the exposure-outcome association.

Note: The models used to generate binary exposure X and binary outcome Y are respectively $\text{logit}(X) = \eta_0 + \eta_1 C_1 + \eta_2 C_2$ and $\text{logit}(Y) = \gamma_0 + \gamma_1 C_1 + \gamma_2 C_2 + \beta X$, where confounders $C_1 \sim Bin(1, 0.1)$ and $C_2 \sim Bin(1, 0.6)$. The default parameter settings here assume strong confounder effects: Setting I $\eta_1 = \eta_2 = \gamma_1 = \gamma_2 = 2$; Setting II $\eta_1 = \eta_2 = \gamma_1 = 2, \gamma_2 = -2$; and Setting III $\eta_1 = \eta_2 = 2, \gamma_1 = \gamma_2 = -2$. Only Setting I "weak" assumes weak confounder effects: $\eta_1 = \eta_2 = \gamma_1 = \gamma_2 = 0.5$.

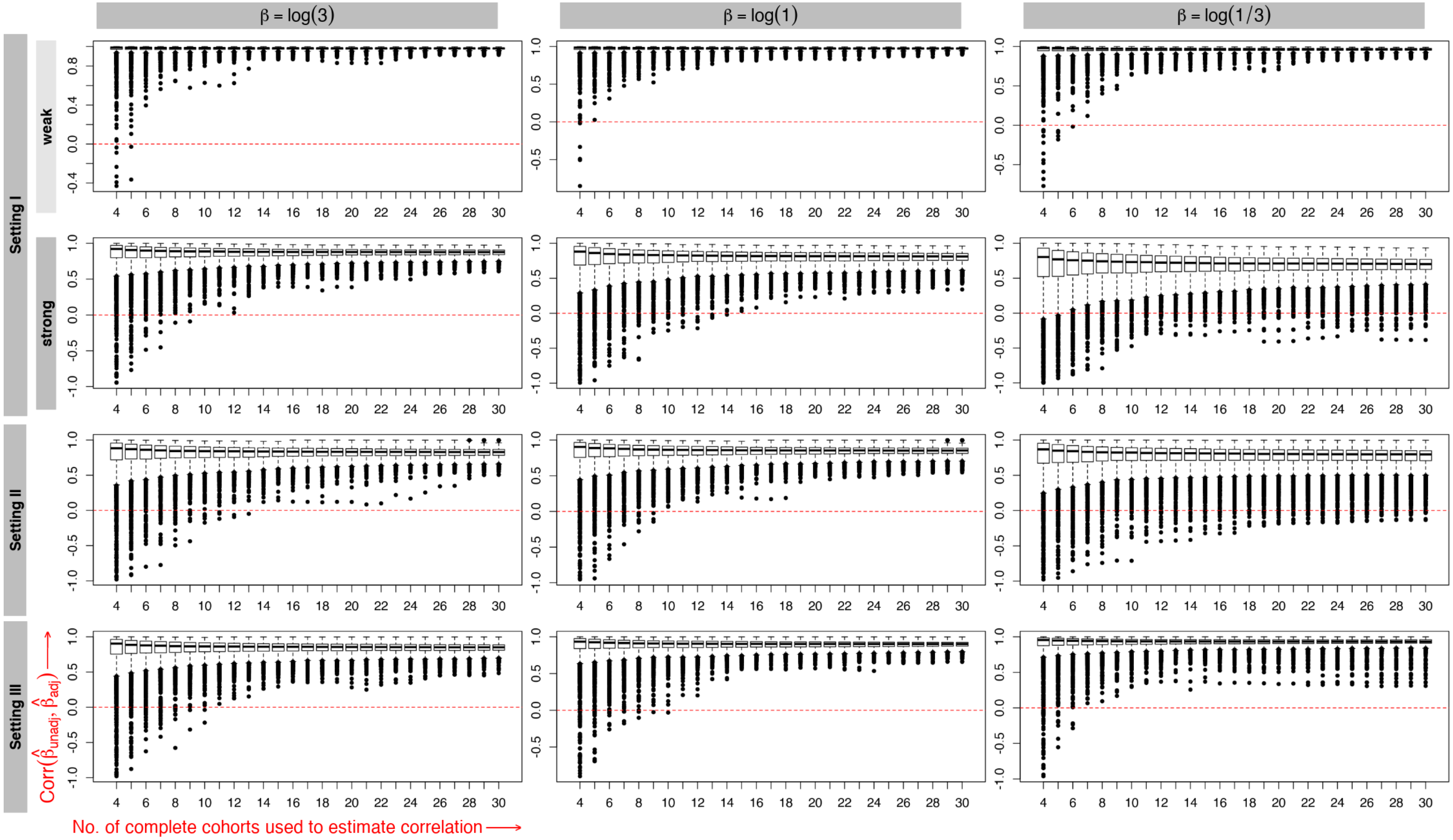

Figure S6: Influence of per-cohort sample size as well as the number of cohorts with complete confounder information on estimated correlation coefficient between unadjusted and adjusted effect estimates. Sample size for each cohort is 300 in one scenario, 150 in another, and much smaller at 30 in yet another scenario. The distribution of estimated $\left(\hat{\beta}_{\text{unadj}}, \hat{\beta}_{\text{adj}}\right)$ is over 2,500 independent replicate datasets for each simulation scenario. The number of complete cohorts needed to estimate this covariance depends on the strengths and directions of the confounder effects as well as the exposure-outcome association.
Note: The models used to generate binary exposure X and binary outcome Y are respectively $\text{logit}(X) = \eta_0 + \eta_1 C_1 + \eta_2 C_2$ and $\text{logit}(Y) = \gamma_0 + \gamma_1 C_1 + \gamma_2 C_2 + \beta X$, where confounders $C_1 \sim Bin(1, 0.1)$ and $C_2 \sim Bin(1, 0.6)$. The parameters for Setting I "weak" assumes weak confounder effects: $\eta_1 = \eta_2 = \gamma_1 = \gamma_2 = 0.5$.

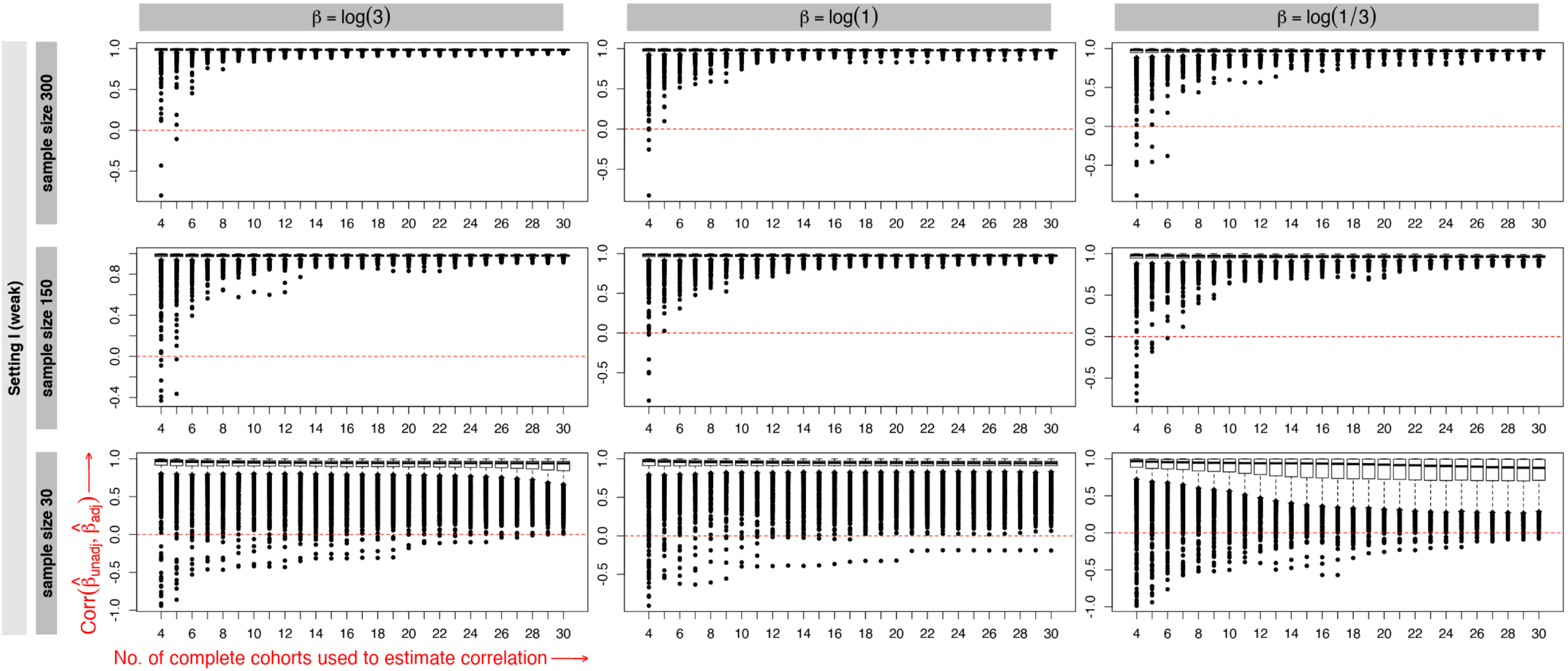

Supplementary S4

Additional proofs, figures and tables.

Proof of Result 3. We begin by noting that here $\hat{\beta}_{\text{unadj}}$ and $\hat{\beta}_{\text{adj}}$ are the maximum likelihood estimates of exposure-outcome association from the unadjusted and the fully adjusted generalized linear models respectively. Under certain regularity conditions (including identifiability, smoothness, and boundedness conditions), large sample theory says that $\hat{\beta}_{\text{unadj}}$ and $\hat{\beta}_{\text{adj}}$ have asymptotic normal distributions (see classic textbooks such as Ferguson (1996), Lehmann (1999), DasGupta (2008)). In particular, if β_{unadj} and β_{adj} are the true population parameters in the unadjusted and the adjusted likelihood functions respectively, then

$$\sqrt{n}\left(\hat{\beta}_{\text{unadj}} - \beta_{\text{unadj}}\right) \xrightarrow{\mathcal{L}} N\left(0, \mathcal{I}^{-1}(\beta_{\text{unadj}})\right) \text{ as } n \to \infty$$
$$\sqrt{n}\left(\hat{\beta}_{\text{adj}} - \beta_{\text{adj}}\right) \xrightarrow{\mathcal{L}} N\left(0, \mathcal{I}^{-1}(\beta_{\text{adj}})\right) \text{ as } n \to \infty$$

where $\xrightarrow{\mathcal{L}}$ denotes convergence in law (or convergence in distribution) and $\mathcal{I}(.)$ is the Fisher's information. Thus, one can write

$$\hat{\beta}_{\text{unadj}} = \beta_{\text{unadj}} + \frac{1}{\sqrt{n}} Z_u, \text{ where } Z_u \sim N\left(0, \mathcal{I}^{-1}(\beta_{\text{unadj}})\right)$$
$$\hat{\beta}_{\text{adj}} = \beta_{\text{adj}} + \frac{1}{\sqrt{n}} Z_a, \text{ where } Z_a \sim N\left(0, \mathcal{I}^{-1}(\beta_{\text{adj}})\right)$$

Consequently, as $n \to \infty$, the bias $\hat{\beta}_{\text{unadj}} - \hat{\beta}_{\text{adj}} \xrightarrow{\text{P}} \text{E}(\hat{\beta}_{\text{unadj}} - \hat{\beta}_{\text{adj}}) = \beta_{\text{unadj}} - \beta_{\text{adj}}$, which is a constant independent of sample size n. ■

Proof of Result 4. In the context of CIMBAL, we have two sets of estimates: $\left(\tilde{\beta}_{1,\text{adj}}, \tilde{\text{se}}_{1,\text{adj}}\right)$ corresponding to cohort 1 with no confounder information and $\left(\hat{\beta}_{2,\text{adj}}, \hat{\text{se}}_{2,\text{adj}}\right)$ corresponding to cohort 2 with full confounder information. Although cohorts 1 and 2 are independent, the CIMBAL-imputed estimate $\tilde{\beta}_{1,\text{adj}}$ and the fully adjusted estimate $\hat{\beta}_{2,\text{adj}}$ are correlated due to borrowing of information between cohorts. In particular,

$$\text{Cov}_b(\tilde{\beta}_{1,\text{adj}}, \hat{\beta}_{2,\text{adj}}) = \text{Cov}_b(\hat{\beta}_{2,\text{adj}} - \hat{\beta}_{2,\text{unadj}} + \hat{\beta}_{1,\text{unadj}}, \hat{\beta}_{2,\text{adj}}) = \text{Var}(\hat{\beta}_{2,\text{adj}}) - \text{Cov}(\hat{\beta}_{2,\text{unadj}}, \hat{\beta}_{2,\text{adj}})$$

where $\text{Cov}_b(.)$ denotes between-cohort covariance, to differentiate from Cov(.) that captures within-cohort co-variability. To meta-analyze adjusted estimates from cohorts 1 and 2, we find the linear combination with the smallest asymptotic variance among all linear estimators of the common exposure-outcome association effect. We consider linear combinations of the form $w_1\tilde{\beta}_{1,\text{adj}} + w_2\hat{\beta}_{2,\text{adj}}$ and, assuming $w_1 + w_2 = 1$, attempt to minimize its variance:

$$\begin{aligned}\text{Var}\left(w_1\tilde{\beta}_{1,\text{adj}} + (1-w_1)\hat{\beta}_{2,\text{adj}}\right) &= \text{Var}\left(\hat{\beta}_{2,\text{adj}} + w_1(\hat{\beta}_{1,\text{unadj}} - \hat{\beta}_{2,\text{unadj}})\right) \\ &= w_1^2\left(\hat{\text{se}}^2_{1,\text{unadj}} + \hat{\text{se}}^2_{2,\text{unadj}}\right) - 2w_1\text{Cov}(\hat{\beta}_{2,\text{unadj}}, \hat{\beta}_{2,\text{adj}}) + \hat{\text{se}}^2_{2,\text{adj}}\end{aligned}$$

Since $\hat{\text{se}}^2_{1,\text{unadj}} + \hat{\text{se}}^2_{2,\text{unadj}} > 0$, a unique minimizer of this variance exists at $\hat{w}_1 = \frac{\text{Cov}(\hat{\beta}_{2,\text{unadj}},\hat{\beta}_{2,\text{adj}})}{\hat{\text{se}}^2_{1,\text{unadj}}+\hat{\text{se}}^2_{2,\text{unadj}}}$ and the minimum variance is $\hat{\text{se}}^2_{2,\text{adj}} - \frac{\text{Cov}(\hat{\beta}_{2,\text{unadj}},\hat{\beta}_{2,\text{adj}})^2}{\hat{\text{se}}^2_{1,\text{unadj}}+\hat{\text{se}}^2_{2,\text{unadj}}}$.

[Recall elementary calculus: a quadratic polynomial $ax^2 + bx + c$ has a unique minimum iff $a > 0$, in which case the minimum occurs at $x = -\frac{b}{2a}$ and the minimum value is $c - \frac{b^2}{4a}$.]

∎

Figure S7: Comparison of CIMBAL with complete case meta-analysis approach and gold standard (oracle) approach for parameter Setting I with weak confounder effects and across different simulation scenarios. The log-odds estimate of the exposure-outcome association ($\hat{\beta} = \log(\hat{\text{OR}})$) and its SE $\left(\hat{\text{SE}} = \sqrt{\hat{\text{Var}}(\hat{\beta})}\right)$ from the combined cohort over 2500 independent replicate datasets are plotted for each scenario: (1) fewer cohorts or (2) equal number of cohorts or (3) more cohorts with no confounder information than with complete confounder information. The horizontal dashed line in the $\hat{\beta}$-plots correspond to the true $\beta = 0$.
Note: The models used to generate binary exposure X and binary outcome Y are respectively $\text{logit}(X) = \eta_0 + \eta_1 C_1 + \eta_2 C_2$ and $\text{logit}(Y) = \gamma_0 + \gamma_1 C_1 + \gamma_2 C_2 + \beta X$, where confounders $C_1 \sim Bin(1, 0.1)$ and $C_2 \sim Bin(1, 0.6)$. Only parameter Setting I with weak confounder effects ($\eta_1 = \eta_2 = \gamma_1 = \gamma_2 = 0.5$) are considered here.

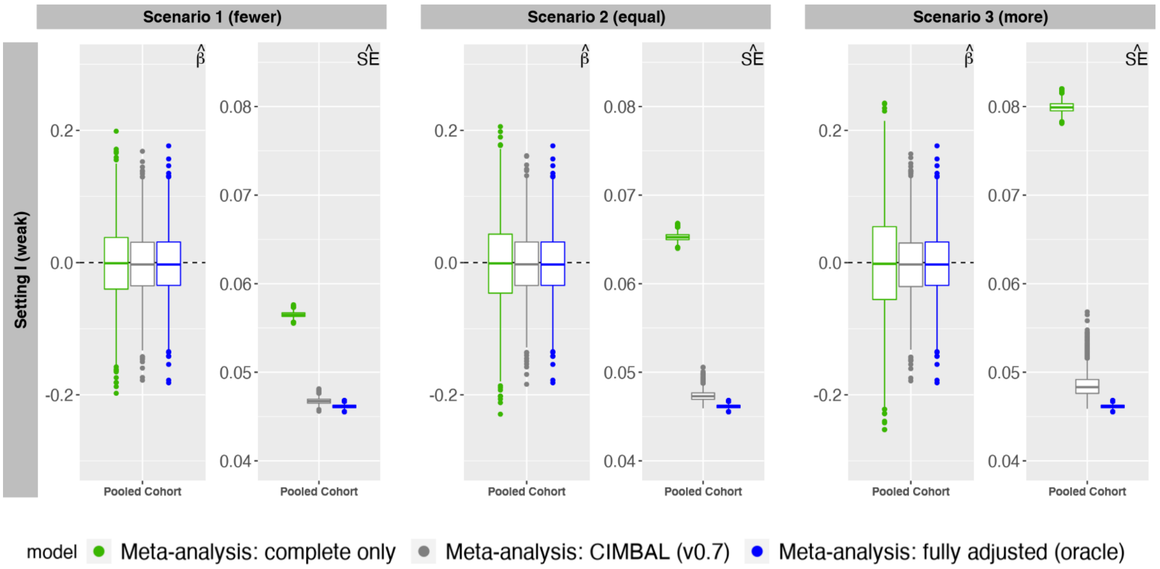

Figure S8: Comparison of CIMBAL with complete case meta-analysis approach and gold standard (oracle) approach for parameter Setting I and across different simulation scenarios. The log-odds estimate of the exposure-outcome association ($\hat{\beta} = \log(\hat{\text{OR}})$) and its SE $\left(\hat{\text{SE}} = \sqrt{\hat{\text{Var}}(\hat{\beta})}\right)$ from the combined cohort over 2500 independent replicate datasets are plotted for each scenario: (1) fewer cohorts or (2) equal number of cohorts or (3) more cohorts with no confounder information than with complete confounder information. The horizontal dashed line in the $\hat{\beta}$-plots correspond to the true β. Regardless of the true association, meta-analysis using CIMBAL is closer to the oracle than other meta-analysis approaches across different scenarios.
Note: The models used to generate binary exposure X and binary outcome Y are respectively $\text{logit}(X) = \eta_0 + \eta_1 C_1 + \eta_2 C_2$ and $\text{logit}(Y) = \gamma_0 + \gamma_1 C_1 + \gamma_2 C_2 + \beta X$, where confounders $C_1 \sim Bin(1, 0.1)$ and $C_2 \sim Bin(1, 0.6)$. All parameter settings here assume strong confounder effects: Setting I $\eta_1 = \eta_2 = \gamma_1 = \gamma_2 = 2$; Setting II $\eta_1 = \eta_2 = \gamma_1 = 2, \gamma_2 = -2$; and Setting III $\eta_1 = \eta_2 = 2, \gamma_1 = \gamma_2 = -2$.

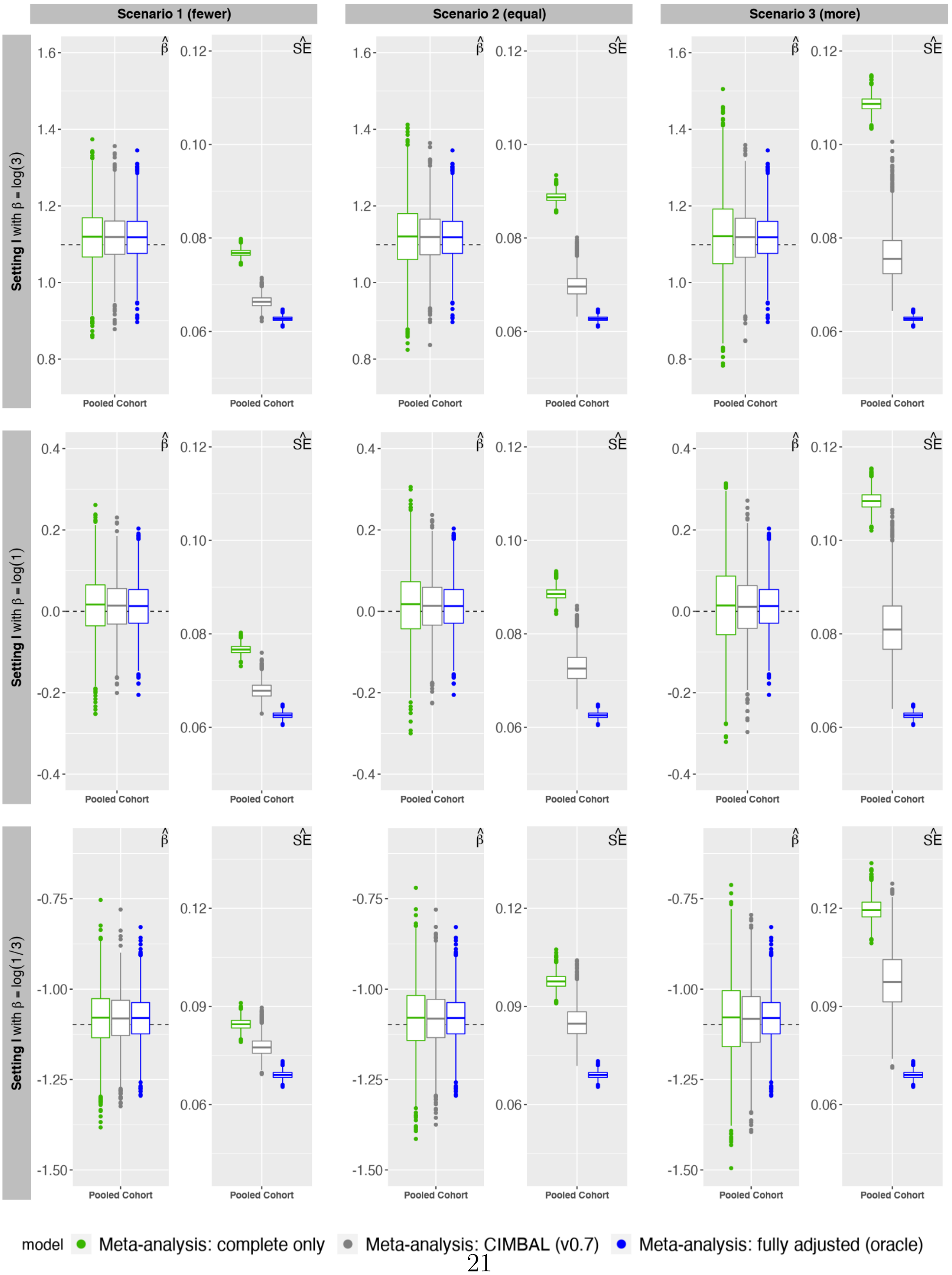

Figure S9: Meta-analysis using CIMBAL boils down to meta-analysis of only complete cohorts when there are not enough complete cohorts to estimate Cov $\left(\hat{\beta}_{\text{unadj}}, \hat{\beta}_{\text{adj}}\right)$. The log-odds estimate of the exposure-outcome association ($\hat{\beta}$) and its SE from the combined cohort over 2500 independent replicate datasets are plotted across different simulation settings. We assume a scenario where 45 out of 60 cohorts have missing confounder information, and deem that 15 complete cohorts are not enough to estimate the covariance, so CIMBAL assumes 0 covariance. The horizontal dashed line in the $\hat{\beta}$-plots correspond to the true $\beta = 0$.
Note: The models used to generate binary exposure X and binary outcome Y are respectively $\text{logit}(X) = \eta_0 + \eta_1 C_1 + \eta_2 C_2$ and $\text{logit}(Y) = \gamma_0 + \gamma_1 C_1 + \gamma_2 C_2 + \beta X$, where confounders $C_1 \sim Bin(1, 0.1)$ and $C_2 \sim Bin(1, 0.6)$. All parameter settings here assume strong confounder effects: Setting I $\eta_1 = \eta_2 = \gamma_1 = \gamma_2 = 2$; Setting II $\eta_1 = \eta_2 = \gamma_1 = 2, \gamma_2 = -2$; and Setting III $\eta_1 = \eta_2 = 2, \gamma_1 = \gamma_2 = -2$.

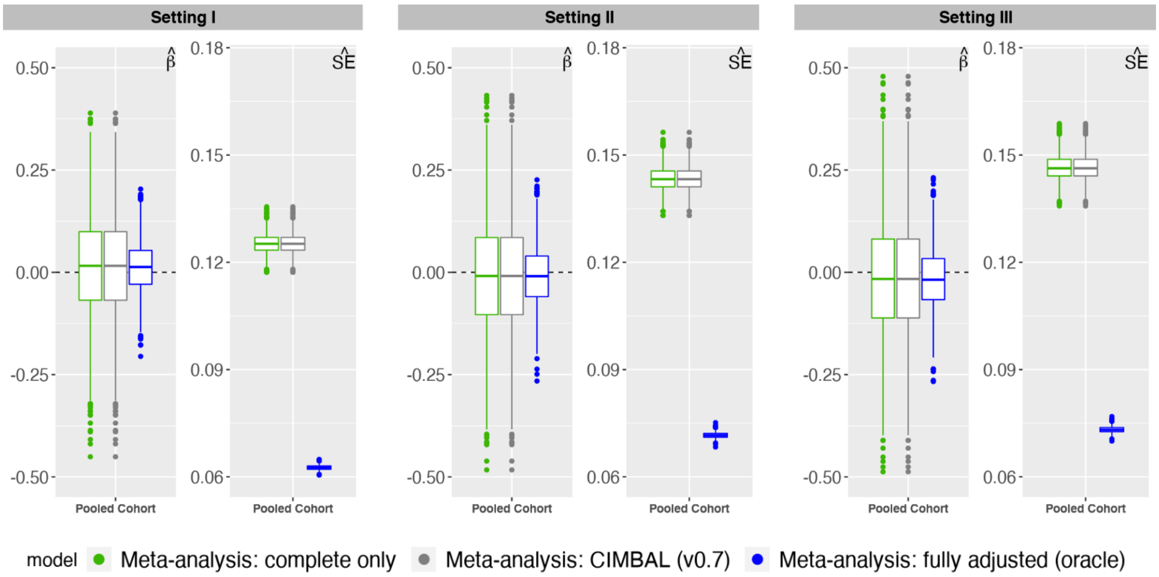

Figure S10: Sensitivity analysis I: Comparison of CIMBAL with complete case meta-analysis approach and gold standard (oracle) approach when the underlying joint distribution is heterogenous. The log-odds estimate of the exposure-outcome association ($\hat{\beta} = \log(\hat{\text{OR}})$) and its SE $\left(\hat{\text{SE}} = \sqrt{\hat{\text{Var}}(\hat{\beta})}\right)$ from the combined cohort over 2500 independent replicate datasets are plotted for each scenario: (1) fewer cohorts or (2) equal number of cohorts or (3) more cohorts with no confounder information than with complete confounder information. The horizontal dashed line in the $\hat{\beta}$-plots correspond to the true $\beta = 0$. Regardless of outcome-confounder and exposure-confounder associations, meta-analysis using CIMBAL is closer to the oracle than other meta-analysis approaches across different scenarios.
Note: The models used to generate binary exposure X and binary outcome Y are respectively $\text{logit}(X) = \eta_0 + \eta_1 C_1 + \eta_2 C_2$ and $\text{logit}(Y) = \gamma_0 + \gamma_1 C_1 + \gamma_2 C_2 + \beta X$. Cohorts with no information on confounders are drawn from a population where confounders $C_1 \sim Bin(1, 0.1)$ and $C_2 \sim Bin(1, 0.6)$. The remaining cohorts (those with complete information) are drawn from a separate population with $C_1 \sim Bin(1, 0.2)$ and $C_2 \sim Bin(1, 0.7)$. This changes the mean as well as the variance of the joint distribution $[Y, X, C_1, C_2]$. All parameter settings here assume strong confounder effects in both populations: Setting I $\eta_1 = \eta_2 = \gamma_1 = \gamma_2 = 2$; Setting II $\eta_1 = \eta_2 = \gamma_1 = 2, \gamma_2 = -2$; and Setting III $\eta_1 = \eta_2 = 2, \gamma_1 = \gamma_2 = -2$.

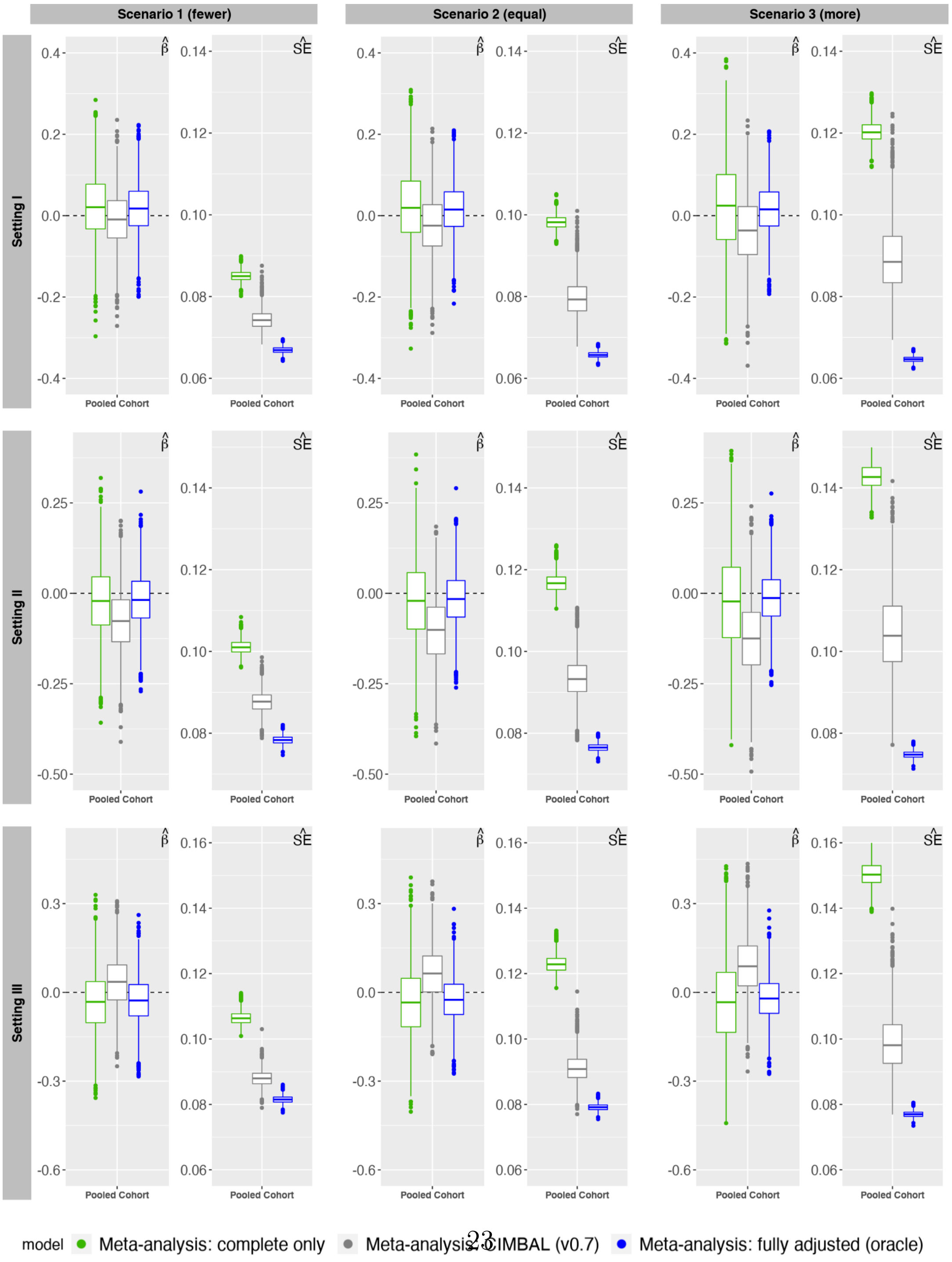

Figure S11: Sensitivity analysis II: Comparison of CIMBAL with complete case meta-analysis approach and gold standard (oracle) approach when the homogeneity of confounding bias across cohorts is violated. The log-odds estimate of the exposure-outcome association ($\hat{\beta} = \log(\hat{\text{OR}})$) and its SE $\left(\hat{\text{SE}} = \sqrt{\hat{\text{Var}}(\hat{\beta})}\right)$ from the combined cohort over 2500 independent replicate datasets are plotted for each scenario: (1) fewer cohorts or (2) equal number of cohorts or (3) more cohorts with no confounder information than with complete confounder information. The horizontal dashed line in the $\hat{\beta}$-plots correspond to the true $\beta = 0$.
Note: The models used to generate binary exposure X and binary outcome Y are respectively $\text{logit}(X) = \eta_0 + \eta_1 C_1 + \eta_2 C_2$ and $\text{logit}(Y) = \gamma_0 + \gamma_1 C_1 + \gamma_2 C_2 + \beta X$, where confounders $C_1 \sim Bin(1, 0.1)$ and $C_2 \sim Bin(1, 0.6)$. Cohorts without any confounder information are drawn from a population where the parameter settings assume strong confounder effects: Setting I $\eta_1 = \eta_2 = \gamma_1 = \gamma_2 = 2$; Setting II $\eta_1 = \eta_2 = \gamma_1 = 2, \gamma_2 = -2$; and Setting III $\eta_1 = \eta_2 = 2, \gamma_1 = \gamma_2 = -2$. The remaining cohorts (those with complete confounder information) are drawn from a separate population where confounding effects are assumed to be considerably weaker: Setting I $\eta_1 = \eta_2 = \gamma_1 = \gamma_2 = 0.5$; Setting II $\eta_1 = \eta_2 = \gamma_1 = 0.5, \gamma_2 = -0.5$; and Setting III $\eta_1 = \eta_2 = 0.5, \gamma_1 = \gamma_2 = -0.5$. This ensures that the joint distributions $[Y, X, C_1, C_2]$ are the same across cohorts but the confounding biases are not.

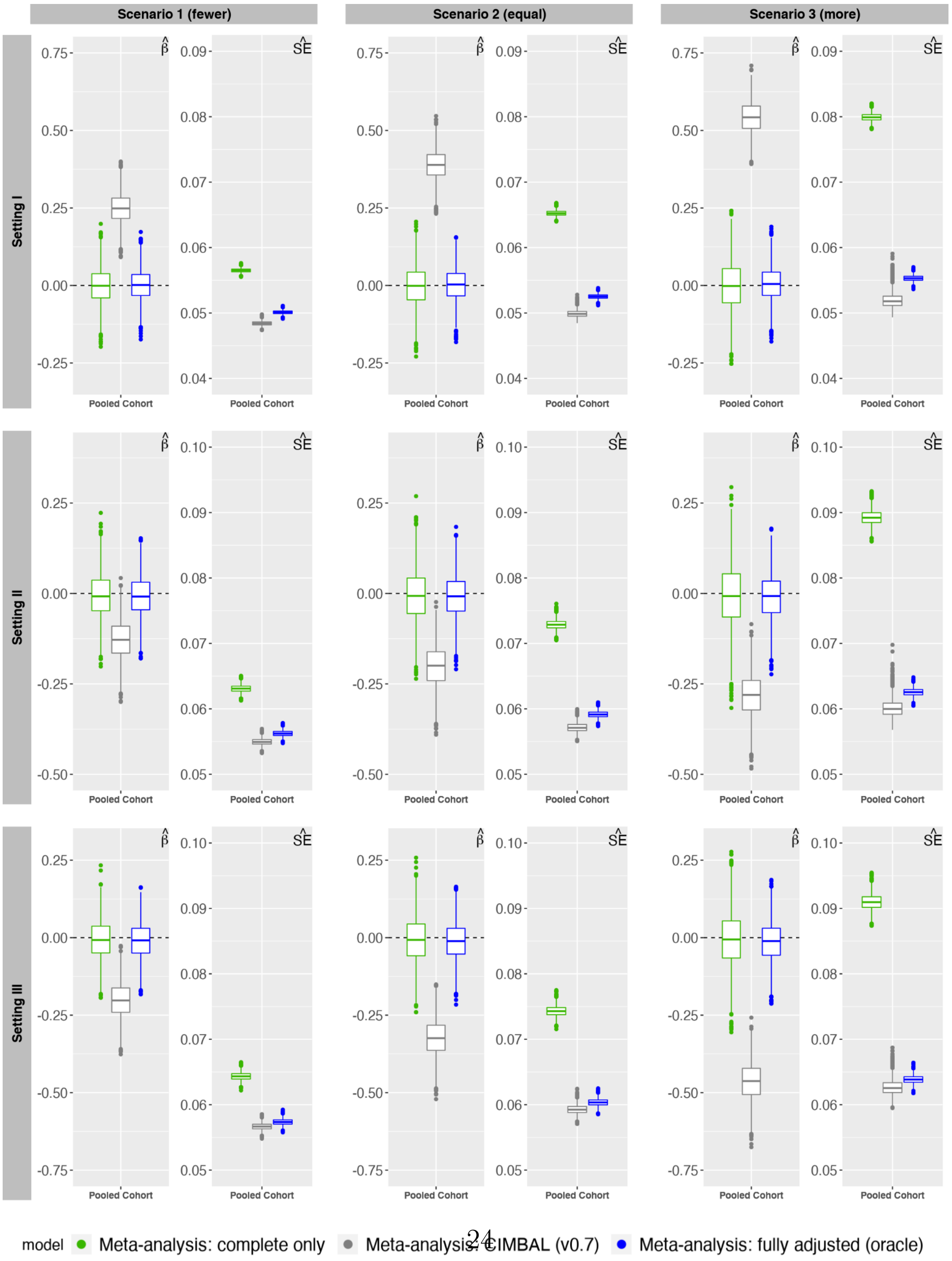

Table S4: Sensitivity analysis I & II: Evaluation of CIMBAL along with complete case meta-analysis approach and gold standard (oracle) approach using multiple metrics across different simulated data scenarios. The metrics MSE (mean squared error), rel. MSE (relative MSE compared to oracle meta-analysis approach), mean width of 95% CI, and type I error inflation factor at 5% significance level (ratio of type I error estimate to 0.05) are estimated using $2,500$ independent replicate datasets for each scenario: (1) fewer cohorts or (2) equal number of cohorts or (3) more cohorts with no confounder information than with complete confounder information. Ideal rel. MSE value is 1× and larger values indicate departure from oracle. Ideal type I error inflation value is 1; larger than 1 indicates inflation, smaller than 1 indicates conservativeness. The underlying data generative model assumes there is no exposure-outcome association (true $\beta = 0$).

		Method	Scenario 1 (fewer)			Scenario 2 (equal)			Scenario 3 (more)		
			MSE (rel. MSE)	mean width	type I error IF	MSE (rel. MSE)	mean width	type I error IF	MSE (rel. MSE)	mean width	type I error IF
**Sensitivity Analysis I**	**Setting I**	M: complete only	0.007 (1.4×)	0.33	0.95	0.009 (2.3×)	0.39	0.89	0.014 (3.5×)	0.47	0.82
		M: CIMBAL (v0.7)	0.005 (1.0×)	0.29	0.86	0.006 (1.5×)	0.31	0.94	0.009 (2.3×)	0.35	1.09
		M: fully adjusted (oracle)	0.005 (1×)	0.26	1.05	0.004 (1×)	0.26	1.08	0.004 (1×)	0.25	1.02
	**Setting II**	M: complete only	0.010 (1.7×)	0.40	1.01	0.013 (2.2×)	0.46	0.86	0.020 (3.3×)	0.56	1.01
		M: CIMBAL (v0.7)	0.013 (2.2×)	0.34	2.90	0.019 (3.2×)	0.37	4.13	0.027 (4.5×)	0.41	4.70
		M: fully adjusted (oracle)	0.006 (1×)	0.31	1.03	0.006 (1×)	0.30	1.06	0.006 (1×)	0.29	1.08
	**Setting III**	M: complete only	0.011 (1.6×)	0.42	1.02	0.015 (2.1×)	0.48	0.93	0.022 (3.7×)	0.59	0.99
		M: CIMBAL (v0.7)	0.009 (1.3×)	0.34	1.27	0.012 (1.7×)	0.36	1.93	0.018 (3.0×)	0.39	2.97
		M: fully adjusted (oracle)	0.007 (1×)	0.32	1.18	0.007 (1×)	0.31	1.16	0.006 (1×)	0.30	1.12
**Sensitivity Analysis II**	**Setting I**	M: complete only	0.003 (1.0×)	0.22	0.97	0.004 (1.3×)	0.26	0.95	0.006 (2.0×)	0.31	0.94
		M: CIMBAL (v0.7)	0.064 (21.3×)	0.19	20.0	0.154 (51.3×)	0.20	20.0	0.296 (98.7×)	0.20	20.0
		M: fully adjusted (oracle)	0.003 (1×)	0.20	0.99	0.003 (1×)	0.21	1.03	0.003 (1×)	0.22	1.06
	**Setting II**	M: complete only	0.004 (1.3×)	0.25	1.05	0.005 (1.3×)	0.29	0.98	0.008 (2.0×)	0.35	0.98
		M: CIMBAL (v0.7)	0.019 (6.3×)	0.22	12.7	0.043 (10.8×)	0.22	18.7	0.083 (20.8×)	0.24	20.0
		M: fully adjusted (oracle)	0.003 (1×)	0.22	1.06	0.004 (1×)	0.23	1.01	0.004 (1×)	0.25	0.97
	**Setting III**	M: complete only	0.004 (1.3×)	0.25	1.14	0.005 (1.3×)	0.29	1.06	0.008 (2.0×)	0.36	0.92
		M: CIMBAL (v0.7)	0.044 (14.7×)	0.22	18.9	0.108 (27.0×)	0.23	20.0	0.219 (54.8×)	0.25	20.0
		M: fully adjusted (oracle)	0.003 (1×)	0.22	1.01	0.004 (1×)	0.24	1.11	0.004 (1×)	0.25	0.92

Figure S12: Sensitivity analysis III: Comparison of CIMBAL with complete case meta-analysis approach and gold standard (oracle) approach when there is an unmeasured confounder. The log-odds estimate of the exposure-outcome association ($\hat{\beta} = \log(\hat{\text{OR}})$) and its SE $\left(\hat{\text{SE}} = \sqrt{\hat{\text{Var}}(\hat{\beta})}\right)$ from the combined cohort over 2500 independent replicate datasets are plotted for each scenario: (1) fewer cohorts or (2) equal number of cohorts or (3) more cohorts with no confounder information than with complete confounder information. Here, true $\beta = 0$. All meta-analysis approaches are extremely biased when there is an unmeasured strong confounder. However, meta-analysis using CIMBAL is still closer to the oracle than other meta-analysis approaches across different scenarios.
Note: The models used to generate binary exposure X and binary outcome Y are respectively $\text{logit}(X) = \eta_0 + \eta_1 C_1 + \eta_2 C_2 + \eta_3 C_3$ and $\text{logit}(Y) = \gamma_0 + \gamma_1 C_1 + \gamma_2 C_2 + \gamma_3 C_3 + \beta X$, where confounders $C_1 \sim Bin(1, 0.1)$ and $C_2 \sim Bin(1, 0.6)$ are measured while $C_3 \sim N(0, 1)$ is unmeasured in every cohort. All parameter settings here assume strong confounder effects: Setting I $\eta_1 = \eta_2 = \gamma_1 = \gamma_2 = 2, \eta_3 = \gamma_3 = 2$; Setting II $\eta_1 = \eta_2 = \gamma_1 = 2, \gamma_2 = -2, \eta_3 = \gamma_3 = 2$; and Setting III $\eta_1 = \eta_2 = 2, \gamma_1 = \gamma_2 = -2, \eta_3 = \gamma_3 = 2$.

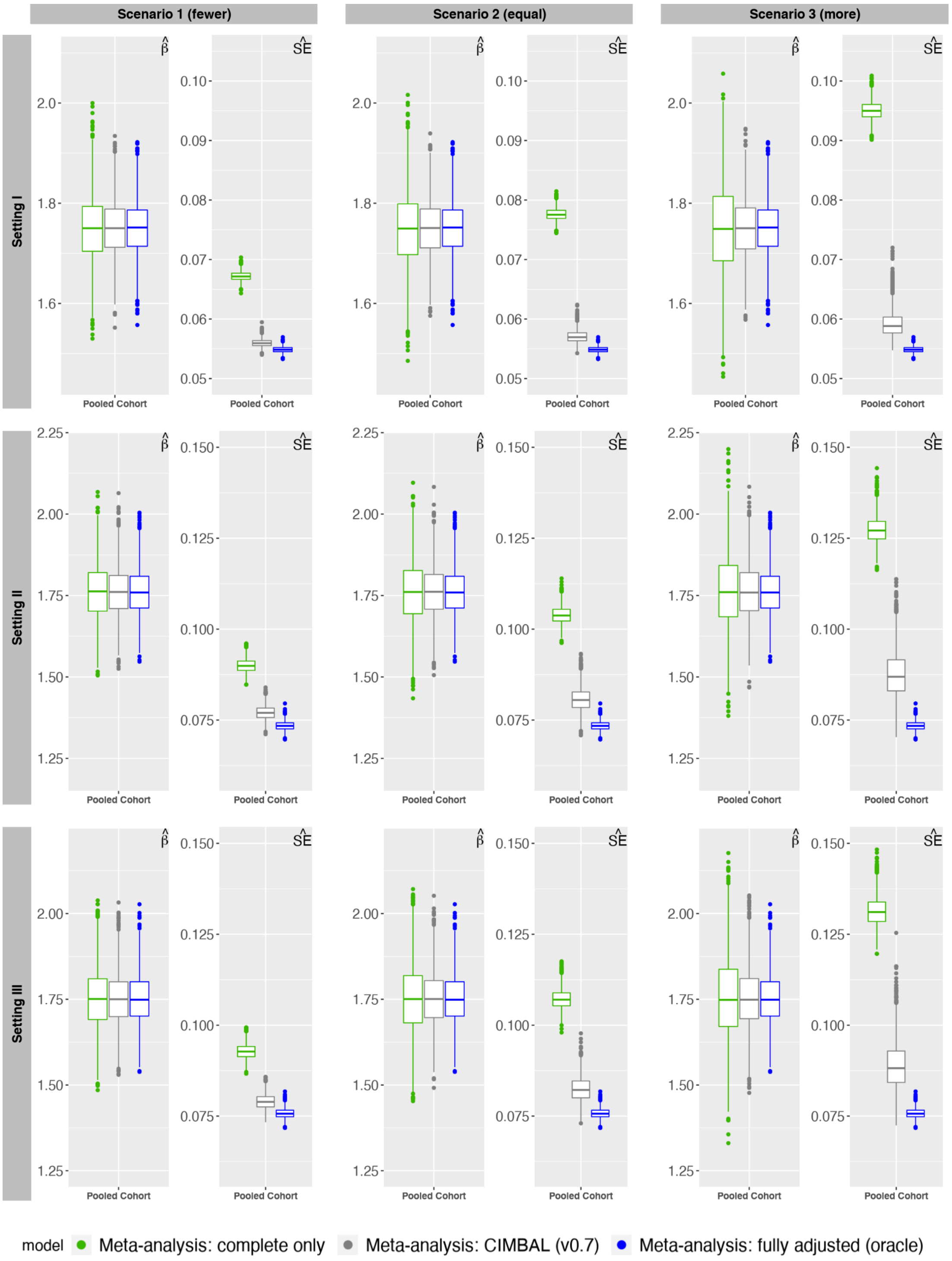

Table S5: Evaluation of CIMBAL and complete case meta-analysis approach with gold standard (oracle) approach using multiple metrics for Setting I with weak confounder effects. The metrics MSE (mean squared error), rel. MSE (relative MSE compared to oracle meta-analysis approach), mean width of 95% confidence interval, and type I error inflation factor at 5% significance level (ratio of type I error estimate to 0.05) are estimated using $2,500$ independent replicate datasets for each scenario: (1) fewer cohorts or (2) equal number of cohorts or (3) more cohorts with no confounder information than with complete confounder information. Ideal rel. MSE value is $1\times$ and larger values indicate departure from oracle. Ideal type I error inflation value is 1; larger than 1 indicates inflation, smaller than 1 indicates conservativeness.

Note: The models used to generate binary exposure X and binary outcome Y are respectively $\text{logit}(X) = \eta_0 + \eta_1 C_1 + \eta_2 C_2$ and $\text{logit}(Y) = \gamma_0 + \gamma_1 C_1 + \gamma_2 C_2 + \beta X$, where confounders $C_1 \sim Bin(1, 0.1)$ and $C_2 \sim Bin(1, 0.6)$. Setting I here assumes weak confounder effects: $\eta_1 = \eta_2 = \gamma_1 = \gamma_2 = 0.5$ and that there is no exposure-outcome association ($\beta = 0$).

	Method	Scenario 1 (fewer)			Scenario 2 (equal)			Scenario 3 (more)		
		MSE (rel. MSE)	mean width	type I error IF	MSE (rel. MSE)	mean width	type I error IF	MSE (rel. MSE)	mean width	type I error IF
**Setting I** (weak)	M: complete only	0.003 (1.5×)	0.22	0.97	0.004 (2.0×)	0.26	0.95	0.006 (3.0×)	0.31	0.94
	M: CIMBAL (v0.7)	0.002 (1.0×)	0.18	1.11	0.002 (1.0×)	0.19	1.04	0.002 (1.0×)	0.19	1.13
	M: fully adjusted (oracle)	0.002 (1×)	0.18	1.10	0.002 (1×)	0.18	1.10	0.002 (1×)	0.18	1.10

Abbreviations: IF, inflation factor; M, meta-analysis of 60 cohorts

References

[1] Ferguson, T.S. *A course in large sample theory.* Texts in Statistical Science Series. Chapman & Hall/CRC, 1996.

[2] Lehmann, E.L. *Elements of large-sample theory.* Springer Texts in Statistics. Springer, New York, NY, 1999.

[3] DasGupta, A. *Asymptotic theory of statistics and probability.* Springer Texts in Statistics. Springer, New York, NY, 2008.
